# Human cardiac myosin–binding protein C restricts actin structural dynamics in a cooperative and phosphorylation-sensitive manner

**DOI:** 10.1074/jbc.RA119.009543

**Published:** 2019-09-13

**Authors:** Thomas A. Bunch, Rhye-Samuel Kanassatega, Victoria C. Lepak, Brett A. Colson

**Affiliations:** Department of Cellular and Molecular Medicine, University of Arizona, Tucson, Arizona 85724

**Keywords:** actin, cardiac muscle, contractile protein, phosphorylation, spectroscopy, cardiac myosin–binding protein C (cMyBP-C), motor protein, myofilament, protein kinase A (PKA), structural dynamics

## Abstract

Cardiac myosin–binding protein C (cMyBP-C) is a thick filament-associated protein that influences actin-myosin interactions. cMyBP-C alters myofilament structure and contractile properties in a protein kinase A (PKA) phosphorylation-dependent manner. To determine the effects of cMyBP-C and its phosphorylation on the microsecond rotational dynamics of actin filaments, we attached a phosphorescent probe to F-actin at Cys-374 and performed transient phosphorescence anisotropy (TPA) experiments. Binding of cMyBP-C N-terminal domains (C0–C2) to labeled F-actin reduced rotational flexibility by 20–25°, indicated by increased final anisotropy of the TPA decay. The effects of C0–C2 on actin TPA were highly cooperative (*n* = ∼8), suggesting that the cMyBP-C N terminus impacts the rotational dynamics of actin spanning seven monomers (*i.e.* the length of tropomyosin). PKA-mediated phosphorylation of C0–C2 eliminated the cooperative effects on actin flexibility and modestly increased actin rotational rates. Effects of Ser to Asp phosphomimetic substitutions in the M-domain of C0–C2 on actin dynamics only partially recapitulated the phosphorylation effects. C0–C1 (lacking M-domain/C2) similarly exhibited reduced cooperativity, but not as reduced as by phosphorylated C0–C2. These results suggest an important regulatory role of the M-domain in cMyBP-C effects on actin structural dynamics. In contrast, phosphomimetic substitution of the glycogen synthase kinase (GSK3β) site in the Pro/Ala-rich linker of C0–C2 did not significantly affect the TPA results. We conclude that cMyBP-C binding and PKA-mediated phosphorylation can modulate actin dynamics. We propose that these N-terminal cMyBP-C–induced changes in actin dynamics help explain the functional effects of cMyBP-C phosphorylation on actin-myosin interactions.

## Introduction

The actin-myosin complex undergoes structural transitions from weak (disordered) to strong (ordered) binding states during muscle contraction (1). Force generation involves the myosin light chain domain transition from the lever arm up to lever arm down position and the actin filament transition from high to low levels of rotational motion (2–4). Parallel changes in actin dynamics during the ATPase cycle of myosin are dependent upon whether weak- or strong-binding myosin is present. Weak-binding myosin induces a structural state of actin with rotational dynamics intermediate between free actin and the complex with strongly bound myosin. Strong-binding myosin induces highly cooperative changes (*n* = ∼5) in actin that restrict rotational motion along the actin filament, whereas weak-binding myosin–induced changes are not propagated (*n* = 1) (4). In other words, one molecule of strongly bound myosin can restrict the rotational motion of several monomers along the actin filament, whereas structural effects of weakly bound myosin are confined to only the myosin-bound monomers of the actin filament.

In cardiac muscle, thick and thin filament structure and contractile function are further regulated by cardiac myosin–binding protein C (cMyBP-C). cMyBP-C is a modular thick filament-associated protein located in the C-zone, where actin-myosin crossbridges are found (5). In the sarcomere, cMyBP-C is positioned in ∼8 stripes on each half of the A-band and this ∼43.5-nm spacing (6) corresponds roughly with (*a*) the spacing of adjacent myosin crowns on the thick filament (42.9 nm) and (*b*) the spacing of ∼7 actin monomers in the thin filament (or the length of tropomyosin; 42 nm). cMyBP-C is anchored to the myosin thick filament and its N terminus extends away from the thick filament and binds to either the actin thin filament (7) or the neck region of myosin (subfragment 2) (8). PKA-mediated phosphorylation of cMyBP-C modulates the kinetics of actin-myosin crossbridge cycling by reducing binding to actin (9) or myosin subfragment 2 (10).

The actin-cMyBP-C complex was first observed *in vitro* (11) and later theorized to occur *in situ* based on analyses of low-angle X-ray diffraction patterns in muscle. cMyBP-C was observed to have a slightly longer periodicity than myosin, and this was postulated to be because of its N-terminal domains extending beyond myosin and binding to actin under certain muscle states (12). More recently, electron tomography (13) and domain-specific antibody labeling of endogenous cMyBP-C in cardiac fibers (7) have provided further support of actin–cMyBP-C interactions *in vivo*. The N-terminal domains C0–C2 binding to filamentous actin has been also been extensively studied by cosedimentation assays (9, 14), small-angle X-ray/neutron scattering (15, 16), and laser trap motility assays (17). It remains to be determined which of the proposed N-terminal domain–binding sites are involved in the actin-binding interface (12, 14, 18), and how cMyBP-C binding influences actin structure and dynamics.

In a previous study, we characterized the influence of full-length cMyBP-C (domains C0–C10) on actin intrafilament rotational dynamics. We observed that, similar to myosin, cMyBP-C restricts actin rotational motion (19). PKA treatment relieved these structural effects on actin without dissociation. These studies were complicated by the presence of cMyBP-C C-terminal domains. *In vivo* these domains are bound tightly to the thick filament preventing their interaction with actin. *In vitro*, in the absence of myosin, these C-terminal domains bind actin and perturb actin dynamics (19, 20). Thus, studies of the effects of cMyBP-C N-terminal domains C0–C2 (in the absence of C-terminal domains) on actin structural dynamics are needed to capture physiologically relevant interactions and dynamics of the actin–cMyBP-C complex. The previous study did examine effects of N-terminal domains C0–C4 and C0–C1, which had small effects on restricting actin rotational dynamics and increased rate, as compared with the large effects of full-length or C-terminal C5–C10 on actin to restrict rotational amplitude and decrease rate (19). The small effects of the N-terminal domains could be complicated by the fact that the truncation of cMyBP-C proteins (*e.g.* C0–C4) when expressed in baculovirus exhibit dramatically altered phosphorylation states (19), leading to variations in structure and function. Here, we use bacterial expression to ensure that basal phosphorylation levels of all cMyBP-C fragments is zero. Finally, the past work used mouse cMyBP-C and here we use human cMyBP-C.

**Figure 1.**
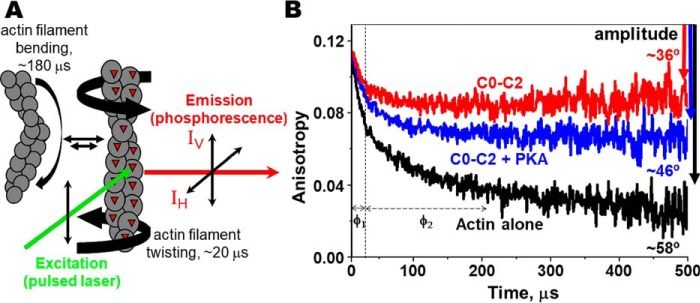
**Transient phosphorescence anisotropy (TPA) of labeled actin filaments.** *A*, actin filament rotational/twisting (and bending) motions in the microsecond time range are detected by TPA. *B*, effect of 4 μm C0–C2 (*red*) or PKA-phosphorylated C0–C2 (*blue*) on the TPA decay of 1 μm ErIA-actin (*black*). The vertical *dotted line* separates the two exponentials of fits for correlation times, φ_1_ (fast mode, twisting motion, ∼20 μs) and φ_2_ (slow mode, bending motion, ∼180 μs). Total change in anisotropy (amplitude) is indicated by the *downward arrows*. The calculated angular amplitude of actin (from Equation 3) is shown. Further details are provided in “Experimental procedures.”

In the present study, we measured transient phosphorescence anisotropy (TPA)^2^ of actin in the presence and absence of N-terminal cMyBP-C through the use of F-actin labeled at Cys-374 with a phosphorescent dye, erythrosine-5-iodoacetamide (ErIA) (Fig. 1*A*). We hypothesized that actin exhibits restricted dynamics when in complex with unphosphorylated C0–C2, and this is reduced significantly when it is complexed with phosphorylated C0–C2. Phosphorylation acts as a regulatory switch. Effects of C0–C2 with phosphomimetic substitutions at the three PKA sites in M-domain or at the glycogen synthase kinase (GSK3β) site in the Pro/Ala-rich (P/A) linker and C0–C1 which lacks M-domain/C2 were also measured using the TPA approach to determine their effects on actin rotational dynamics.

## Results

### Binding of N-terminal cMyBP-C to ErIA-actin

**Figure 2.**
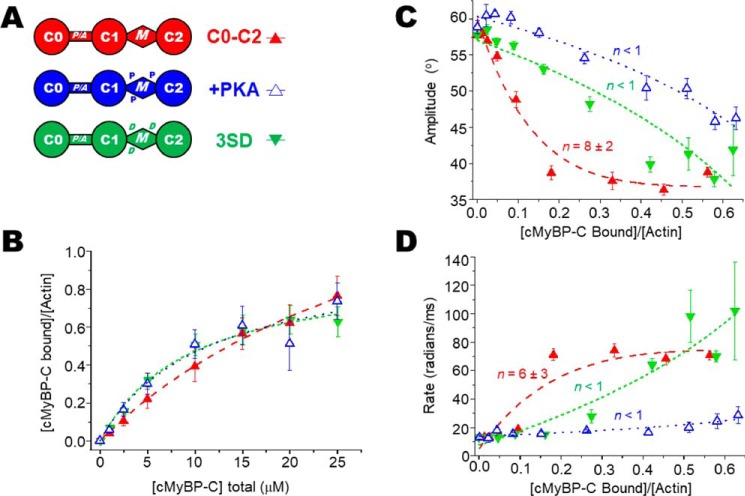
**Effects of PKA phosphorylation and phosphomimetic substitutions in M-domain of C0–C2 on the TPA of actin.** *A*, domain organization of N-terminal cMyBP-C constructs C0–C2, C0–C2+PKA, and C0–C2 3SD. Domains listed from N-terminal C0 to C2, including the Pro/Ala-rich linker (P/A) and M-domain containing three phosphorylation sites (the P in +PKA) and replacement of the phosphorylated serines with aspartic acid residues (the D in 3SD). *B*, binding, from cosedimentation experiments, of C0–C2 constructs to 1 μm phalloidin-stablized ErIA-actin. *C*, angular amplitude (°) was calculated from final anisotropy of actin (Fig. 1*B* and Equation 3) as a function of *v*, the fraction bound to N-terminal cMyBP-C. Curves show fits of a cooperativity model to determine *n* (χ^2^ = 4, 2, and 11 for C0–C2, C0–C2+PKA, and C0–C2 3SD, respectively. For reference, for linear fits χ^2^ = 25, 1, and 5, respectively.). *D*, rates of rotational motions (radians/ms) as a function of *v*, the fraction bound, and fit to a cooperativity model to determine *n. Error bars* denote ± S.E.

**Figure 3.**
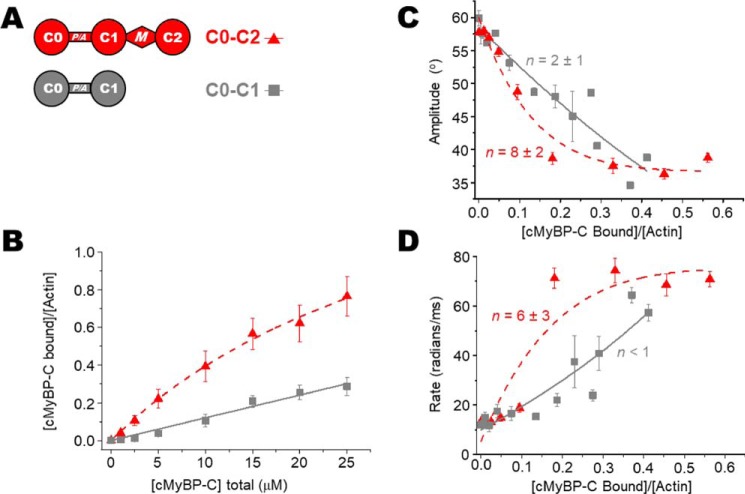
**Effects of C0–C1 (lacking M-domain) on the TPA of actin.** *A*, domain organization of N-terminal cMyBP-C constructs C0–C2 and C0–C1. *B*, binding, from cosedimentation experiments, of C0–C2/C0–C1 constructs to 1 μm phalloidin-stabilized ErIA-actin. *C*, angular amplitude (°) of actin as a function of *v*, the fraction bound to N-terminal cMyBP-C. Curves show fits of a cooperativity model to determine *n* (χ^2^ = 4 and 6 for C0–C2 and C0-C1, respectively. For reference, for linear fits χ^2^ = 25 and 6, respectively.). *D*, rates of rotational motions (radians/ms) as a function of *v*, the fraction bound and fit to a cooperativity model to determine *n. Error bars* denote ± S.E.

**Figure 4.**
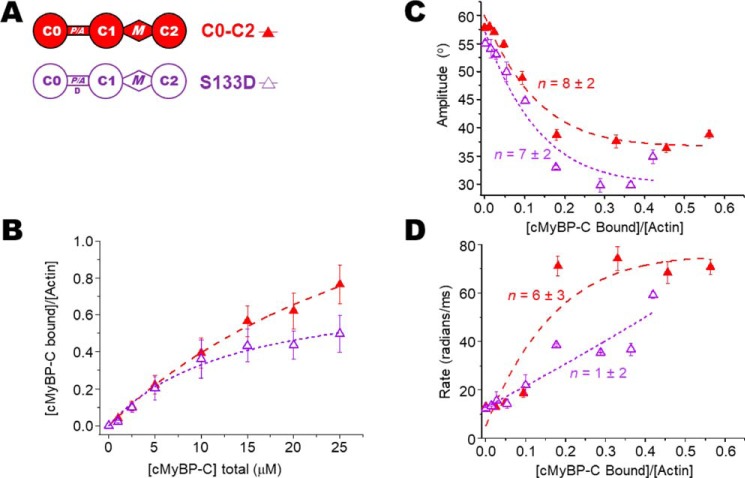
**Effects of S133D mutation to mimic P/A linker phosphorylation on the TPA of actin.** *A*, domain organization of N-terminal cMyBP-C constructs C0–C2 and C0–C2 S133D. The Asp for Ser substitution (*D*) is at the putative GSK3β phosphorylation site in the P/A linker. *B*, binding of C0–C2 constructs to 1 μm phalloidin-stablized ErIA-actin. *C*, angular amplitude (°) of actin as a function of *v*, the fraction bound to N-terminal cMyBP-C. Curves show fits of a cooperativity model to determine *n* (χ^2^ = 4 and 9 for C0–C2 and C0–C2 S133D, respectively. For reference, for linear fits χ^2^ = 25 and 27, respectively.). *D*, rates of rotational motions (radians/ms) as a function of *v*, the fraction bound, and fit to a cooperativity model to determine *n. Error bars* denote ± S.E.

To determine the ratios of cMyBP-C to actin in the TPA experiments, we measured binding of N-terminal cMyBP-C variants (see Figs. 2*A*, 3*A*, and 4*A*) to 1 μm phalloidin-stabilized ErIA-actin by cosedimentation under the same conditions as in our TPA experiments (see Figs. 2*B*, 3*B*, and 4*B*). For comparison with previous studies, the affinity of N-terminal cMyBP-C constructs for 5 μm unlabeled actin was also determined (see Fig. 6, *A* and *D*). Apparent dissociation constants (*K_d_*) and molar binding ratios (*B*_max_) were in the range of affinities reported previously (9, 21–23). At 5 μm, labeling of Cys-374 of actin with ErIA, followed by stabilization with phalloidin, did not change the cosedimentation with N-terminal cMyBP-C (not shown). More detailed comparison of cosedimentation binding curves done at 1 *versus* 5 μm F-actin demonstrated that we were not completely justified in extrapolating data from cosedimentation at 5 μm actin to binding at 1 μm actin. Fig. S1 shows detailed binding comparison of 1 μm ErIA-labeled phalloidin-stabilized F-actin *versus* 5 μm unlabeled F-actin for C0–C2 and C0–C1 constructs. We note that the effects of C0–C2 on actin TPA saturate below 10 μm C0–C2, where the 1 μm actin-binding curve for C0–C2 is similar to that extrapolated from binding at 5 μm actin (Fig. S1*A*). In contrast, effects of C0–C1 on TPA saturate above 10 μm C0–C1 and the 1 μm actin-binding curve for C0–C1 is significantly different from that extrapolated from binding at 5 μm at all concentrations (Fig. S1*B*). Therefore, we have used only the data from 1 μm actin cosedimentation in determining ratios of cMyBP-C to actin in the TPA experiments.

### Effects of C0–C2 and PKA phosphorylation of M-domain on the TPA of actin

C0–C2 affects the TPA of actin and this is modified by PKA phosphorylation of the M-domain (Fig. 1). The effect of unphosphorylated C0–C2 binding to actin is indicated by the change in maximum anisotropy from *r*_∞_(actin alone) = 0.024 ± 0.003 to *r*_∞_(C0–C2) = 0.089 ± 0.003. This effect is significantly greater than *r*_∞_(C0–C2+PKA) = 0.057 ± 0.003. Converting the *r*_∞_ to angular amplitudes (see Equation 3) yields rotational flexibilities of 58°, 46°, and 36° for actin alone, actin plus phosphorylated C0–C2, and actin plus unphosphorylated C0–C2, respectively (Fig. 1*B*). These data indicate that actin bound to phosphorylated C0–C2 assumes a dynamic state intermediate between free and C0–C2 bound states.

The effect on actin's rotational dynamics was measured by TPA over a wide range of C0–C2/actin-binding ratios for unphosphorylated C0–C2, PKA-treated C0–C2, and PKA-phosphomimetic C0–C2 3SD (the three serines phosphorylated by PKA mutated to aspartic acids). Each of these C0–C2 complexes increases the anisostropy, indicating a reduction of actin's angular amplitude (Fig. 2*C*). At all levels of bound C0–C2, the effect of 3SD is significantly less than that of unphosphorylated C0–C2, and the effect of phosphorylation is even more pronounced. Whereas free actin rotates with an amplitude of 58°, unphosphorylated, 3SD, and phosphorylated C0–C2 maximally restrict actin rotation to just 36°, 40°, and 46°, respectively. The maximum standard error of these angular amplitudes is ±1° (Table 1). Fits to the linear lattice model (see Equation 5), as in previous work using actin-binding proteins, myosin (4, 24), and dystrophin (25, 26), show that in addition to differences in the extent of the effects, there are also differences in the cooperativity of the restriction in rotational dynamics. Unphosphorylated C0–C2 was highly cooperative (*n* = 8 ± 2), indicating that binding of one C0–C2 molecule has a spreading effect to eight actin monomers in the filament. This contrasts with the effect of phosphorylated or 3SD mutant C0–C2 where no cooperativity was observed (*n* < 1) (Fig. 2*C* and Table 2). χ^2^ values of the fits indicated that for unphosphorylated C0–C2 (exhibiting cooperativity), the linear lattice model better described the data than a linear function. In contrast, χ^2^ values for fits of proteins with no cooperativity (phosphorylated C0–C2 and C0–C2 3SD) fit similarly well to both models, indicating that a straight line is sufficient to describe the data. We note that the χ^2^ values are above 1, indicating that we do not fully understand the relationship between bound cMyBP-C and effects on actin TPA (Fig. 2*C*).

**Table 1:** **TPA-derived parameters at saturation of effect** Data represent mean ± S.E. (*n* > 4). All bound cMyBP-C significantly reduced amplitude (*p* ≤ 0.0001) and increased the combined rate (*p* ≤ 0.003) at saturation as compared to actin alone. Significant difference in amplitude or combined rate relative to C0–C2 (*, *p* ≤ 0.03; **, *p* ≤ 0.0005; ***, *p* ≤ 0.0001) or C0–C1 (#, *p* ≤ 0.001). Significant difference in correlation times, φ_1_ or φ_2_, as compared to actin alone (#, *p* < 0.03). Saturation of correlation times were determined as the average of all concentrations, but excluding the points denoted by *arrows* in Fig. 5. Note that for two exponential fits the weighting (r_1_, r_2_) of the correlation times (φ_1_, φ_2_) does not differ between actin alone and C0–C2 binding, where r_1_ ∼0.07 and r_2_ ∼0.04.

cMyBP-C	Amplitude	φ_1_	φ_2_	Combined rate
	°	μ*s*	μ*s*	*radians/ms*
Actin alone	58 ± 1	19 ± 1	179 ± 10	13 ± 1
C0–C2	36 ± 1	17 ± 1	167 ± 11	71 ± 4
C0–C2+PKA	46 ± 1***#	15 ± 1#	165 ± 16	29 ± 6**
C0–C2 3SD	40 ± 1*	18 ± 1	184 ± 13	70 ± 4
C0–C2 S133D	33 ± 1	23 ± 1#	157 ± 14	59 ± 1*
C0–C1	39 ± 1	16 ± 1	153 ± 12	57 ± 3*

**Table 2:** **Cooperativity (*n*) of TPA fit parameters of ErIA–actin–cMyBP-C complex** Data represent mean ± S.E. (n > 4). Significant difference in *n* relative to C0–C2 (*, *p* < 0.05).

cMyBP-C	Amplitude	Rate
	°	*ms*^−1^
C0–C2	8 ± 2	6 ± 3
C0–C2+PKA	*n* < 1*	*n* < 1
C0–C2 3SD	*n* < 1*	*n* < 1
C0–C2 S133D	13 ± 4	14 ± 8
C0–C1	2 ± 1*	*n* < 1

### Effects of C0–C1 on the TPA of actin

To begin to assess the contributions of different domains in the N-terminal region of cMyBP-C to effects on actin rotational dynamics, we examined the effects of C0–C1, which lacks both the M-domain and C2. As expected, Fig. 3*B* (and Fig. 6*B*) shows that C0–C2 binds to actin with a higher affinity than C0–C1. To compare the TPA results independently of the different actin-binding affinities, we highlight that we report TPA results as a function of the fractional binding to actin (*v*, mol cMyBP-C bound/mol actin) not as a function of cMyBP-C concentration. C0–C1 maximally restricted the angular amplitude to 39° (Fig. 3*C*). This is less restricted than C0–C2 (36°) but considerably more than C0–C2+PKA (46°) (Table 1). However, C0–C1 effects were not significantly cooperative (*n* = 2 ± 1). To summarize, the magnitude of effects we observe on actin rotational dynamics by TPA are C0–C2 > C0–C1 > C0–C2 3SD > phosphorylated C0–C2. χ^2^ values of the fits indicated that C0–C1, exhibiting little cooperativity, can be sufficiently described by a line and the linear lattice model did not significantly improve the fit (Fig. 3*C*).

### Effects of GSK3β phosphorylation of P/A linker on the TPA of actin

Phosphorylation of cMyBP-C has also been observed in the P/A linker between C0 and C1. It has been suggested that this phosphorylation is mediated by GSK3β. We were unable to phosphorylate C0–C2 *in vitro* even with very high levels of GSK3β. As an alternative approach, we made a phosphomimetic C0–C2 S133D mutant, where the GSK3β site is mutated from Ser to Asp, as depicted in Fig. 3*A*. C0–C2 S133D binding to actin was the same as WT C0–C2 (Fig. 3*B*; see also Fig. 6, *C* and *D*). In contrast to the phosphomimetic 3SD in the motif, the S133D mutation did not reduce cooperativity (*n* = 13 ± 4) nor the effect on angular rotation (Fig. 4 and Table 2). χ^2^ values of the fits indicated that for C0–C2 S133D, exhibiting cooperativity, the linear lattice model better described the data than a linear function (Fig. 4*C*).

### Effects of N-terminal cMyBP-C on fast (twisting) and slow (bending) intrafilament motions

In addition to the angular amplitude and overall rate of actin rotational motions (a combination of both twisting and bending), we analyzed the separate rates of twisting and bending. TPA final amplitude captures the rotational motions of actin, which is largely because of twisting with a very minor component of bending (27). However, TPA rate is particularly sensitive to resolving the timescales of twisting and bending motions. Even though bending is a minor component of the total rotational amplitude, it contributes to the overall rate of the dynamics. This is because of bending of the actin filament that occurs with a very slow rate of <10 radians/ms, whereas the rate of twisting is ∼80 radians/ms (26, 27). Looking at the overall rates (combining twisting and bending) in Figs. 2*D*, 3*D*, and 4*D* and Table 1 suggests that C0–C2 increases the rate of actin dynamics in a cooperative manner and phosphorylation or phosphomimetic mutants do so at a much-reduced level and with no cooperativity. These overall rates are misleading as they are due to averaged changes in the proportion of bending *versus* twisting rates, not the rates of the individual dynamic components.

To separate bending and twisting, we fit the TPA curves to either one or two exponential decays and obtained corresponding correlation times (inverse of the rate). Actin was best fit to two exponentials with correlation times φ_1_ (∼19 μs) reporting the fast mode of twisting and φ_2_ (∼180 μs) attributed to bending. Actin bound to C0–C2 at *v* < 0.1 fit best to two exponentials with φ_1_ (∼17 μs) reporting the fast mode of twisting and φ_2_ (∼170 μs), which are not significantly different from actin alone. When actin was bound to C0–C2 at *v* > 0.1 the TPA fit best to a single exponential with a similar φ_1_. At *v* > 0.1, C0–C2 did not significantly increase twisting rate nor did it increase the bending rate. It removed the slow bending component, resulting in the apparent increase in overall rate that is observed in Fig. 2*D* and Table 1.

**Figure 5.**
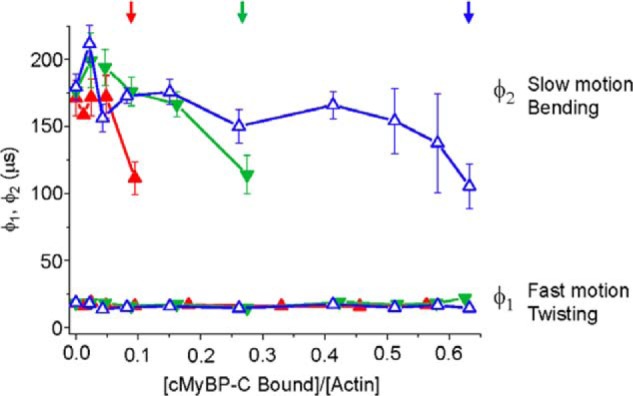
**Analysis of μs correlation times.** The effects of increasing fractions (*v*) of bound C0–C2 (unphosphorylated (*red*), 3SD (*green*), and PKA-phosphorylated (*blue*)) on the individual correlation times for φ_1_, the fast mode that captures twisting motions, and φ_2_, the slow mode that captures bending motions. Note that correlation time is inversely proportional to rate (Equation 4). *Arrows* indicate the highest binding ratio *v* of (cMyBP-C bound)/(actin) at which the TPA decays fit best to two exponentials. Higher binding ratios fit best to one exponential—the fast, twisting, motion. *Error bars* denote ± S.E.

Fig. 5 and Table 1 show the φ_1_ (twisting) and φ_2_ (bending) correlation times for increasing binding of unphosphorylated, 3SD, and phosphorylated C0–C2. Unphosphorylated and the 3SD mutant did not change either φ_1_ or φ_2_ times. In these cases, the increases in overall TPA rate to ∼60–70 radians/ms because of N-terminal cMyBP-C binding (Table 1) is not caused by an increase in rotational dynamics *per se*, but rather caused by a loss of actin bending motions, which occurs at saturation of the TPA effects.

Phosphorylated C0–C2 and C0–C1 showed a modest reduction in φ_1_ as compared with actin alone, corresponding to an increased rate of the twisting from ∼80 to 110 radians/ms. The φ_2_ correlation time was not significantly different from actin alone, corresponding to a rate of bending of ∼9–10 radians/s. To summarize effects on rates of actin motions, unphosphorylated C0–C2 does not affect twisting or bending rates, but it does inhibit bending, whereas phosphorylated C0–C2 modestly increases twisting rates.

We also note that one and two exponential fits give the same results for final anisotropy. That is, the rotational angular magnitude is due primarily to only one component (twisting motions within the actin filament), as described previously (27).

### cMyBP-C phosphorylation analysis and site localization by LC-MS/MS

PKA readily phosphorylated C0–C2 as demonstrated by Pro-Q Diamond staining of treated protein. GSK3β showed no phosphorylation even though it was present at 100× levels (see “Experimental procedures” for details). As not all phosphorylation sites are equally detected by Pro-Q Diamond, we performed microcapillary LC-MS/MS phosphorylation analysis (28) on the kinase-treated proteins. Three serines (Ser-275, Ser-284, and Ser-304) in M-domain (between C1 and C2) were identified as being phosphorylated by PKA. Peptides containing the fourth potential PKA target serine (Ser-311) were detected only as nonphosphorylated (<0.2% phosphorylated; Table S1 and Fig. S2). Peptides containing the potential GSK3β target serine (Ser-133) were also detected but not phosphorylated.

### cMyBP-C impacts actin intrafilament dynamics rather than bundling

**Figure 6.**
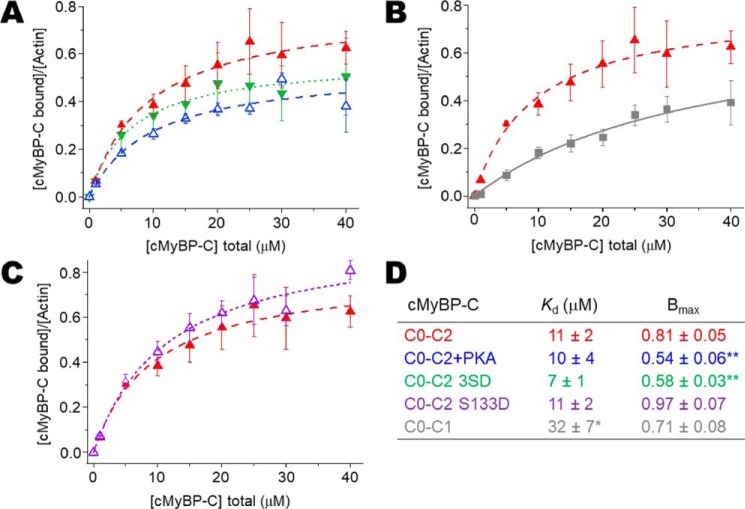
**Cosedimentation binding data for N-terminal cMyBP-C with unlabeled actin at 5 μm.** Although binding data used for TPA analysis were done using phalloidin-stabilized ErIA-labeled F-actin at 1 μm, here nonstabilized and unlabeled F-actin at 5 μm (final concentration) is used to demonstrate that binding of N-terminal cMyBP-C (0–40 μm) with actin under these conditions is consistent with earlier results by others (9, 22, 23). *A–C*, comparison of C0–C2 (in *red* in each panel) with modifications and mutations that were used in Figs. 2, 3, and 4, respectively. *D*, apparent dissociation constants (*K_d_*) and molar binding ratios (*B*_max_) for binding of recombinant N-terminal cMyBP-C to unlabeled F-actin. Data represent mean ± S.E. Significant difference in *K_d_* or *B*_max_ relative to human C0–C2 (*, *p* < 0.01; **, *p* < 0.005). *Error bars* denote ± S.E.

N-terminal cMyBP-C fragments in high stoichiometries have the capacity to tightly bundle actin at physiological pH 7.4; however, bundling is much reduced at pH 8.0 (9). Therefore, all binding and TPA experiments were performed at pH 8.0. To confirm that C0–C2 did not cause actin bundling, we performed two assays for detecting bundling under the conditions of our TPA spectroscopy experiments. First, we monitored the level of turbidity using UV-visible spectroscopy (9) and observed only small increases in turbidity near saturation of the TPA effects. Second, we measured TR-FRET (time-resolved FRET) between F-actin labeled with 5-((((2-iodoacetyl)amino)ethyl)amino)naphtalene-1-sulfonic acid (IAEDANS)- and F-actin labeled with fluorescein-5-maleimide (Fmal) (25). Bundling the actin with 20 mm MgCl_2_ resulted in substantial FRET (35% FRET efficiency; see Equation 6). In contrast to this, C0–C2 at levels where the TPA effect was maximal (5 μm C0–C2 added, *v* = 0.3) (Figs. 2*B* and 6) showed very little difference (1.2% change) in FRET efficiency from the control where no C0–C2 was added (Table 3). Even at the highest concentrations tested of 40 μm C0–C2 there was only a 3% change. We conclude that the C0–C2 impact on actin dynamics, as measured by TPA, reflects the internal dynamics of actin filament, not interactions between filaments.

**Table 3:** **Effects of C0–C2 binding on the FRET efficiency of donor-acceptor labeled F-actin** Data represent mean ± S.E. (n > 4). The positive control (actin induced to bundle with 20 mm MgCl_2_ (0+Mg*)) shows the expected high FRET efficiency (∼35%), calculated from fluorescent lifetimes of donor-only τ_D_ and donor-acceptor τ_DA_ (Equation 6). In contrast, binding of 0–40 μm C0–C2 caused very little change in FRET efficiency (∼3–5%) as compared to actin alone (∼2%).

C0–C2 added	FRET efficiency
μ*m*	*E*
0	1.5 ± 0.1
0+Mg*	34.9 ± 0.1
5	2.7 ± 0.2
10	3.7 ± 0.1
20	5.3 ± 0.1
40	4.4 ± 0.3

## Discussion

The structural effects of interactions between N-terminal cMyBP-C and the actin filament and subsequent impact on cardiac muscle function are a challenge to resolve. This study addresses the impact of cMyBP-C N-terminal domains on F-actin structural dynamics in solution. Determination of cMyBP-C binding to phosphorescently labeled actin under the conditions of our spectroscopy experiments (Figs. 2*B*, 3*B*, and 4*B*) allowed us to calculate accurately the fraction of actin monomers in the F-actin filaments with bound cMyBP-C. This allowed us to determine quantitatively the spectral properties of the bound complex of actin with different mutants or phosphorylation of cMyBP-C (Figs. 2–5 and Table 1).

### Phosphorylation effects on cMyBP-C restriction of actin dynamics

Phosphorylation of C0–C2 dramatically influences the effect that binding has on the microsecond dynamics of actin. Unphosphorylated and phosphorylated C0–C2 bind to actin and restrict its rotational dynamics, but phosphorylated C0–C2 has a smaller effect (12° reduction *versus* 22° reduction in twisting) and cooperativity is removed (Fig. 2*C* and Table 1). Unphosphorylated C0–C2 has a highly cooperative effect on actin, with the effect of a single bound cMyBP-C molecule propagating to approximately eight monomers. Phosphorylated cMyBP-C molecules affect only a single labeled actin monomer. The effects of unphosphorylated C0–C2 are apparently propagated along the actin filaments, because the spectral changes are maximal and saturate at low ratios (1 to ∼8) of cMyBP-C to actin (Fig. 2, *C* and *D*, and Table 2).

These results are consistent with our previously reported TPA measurements showing that the dynamics of actin during interaction with full-length cMyBP-C (C0–C10) are restricted in a cooperative manner when unphosphorylated and much less restricted upon PKA treatment to phosphorylate cMyBP-C (19). Showing that the effects on actin dynamics can be mediated by domains C0–C2 is an important refinement of the earlier work on C0–C10 that suffered from a clear potential complication. C-terminal domains beyond C2 can also bind actin with micromolar affinity (20) and restrict actin dynamics (19). Some of these domains, unlike C0–C2, would not normally come in contact with actin thin filaments in the muscle cell as the cMyBP-C C terminus (C8–C10) is anchored to the myosin thick filament with high affinity (29) and the central domains (C3–C7) are thought to span the inter-thick/thin filament space (7). The effects of C0–C1, C0–C4, and phosphorylated C0–C4 observed in our earlier study (19) were smaller in magnitude than those in the present study. Additionally, the rate for C0–C1 differed from that observed in the current work. We can speculate on the explanation for these differences. The earlier study used baculovirus-expressed cMyBP-C, and it has been shown that the truncation of cMyBP-C to protein fragments in this system (*e.g.* C0–C4) exhibits altered phosphorylation states (30). In the present study we have used cMyBP-C fragments produced in bacteria with no basal phosphorylation. If the fragments used in the previous study were in a partially phosphorylated state then we would expect them to show reduced magnitude of changes in the TPA and a smaller differences upon full phosphorylation. Another possibility for these differences is that the earlier study used mouse and the present study uses human cMyBP-C. We have identified differences in binding actin between the two species, including increased actin binding for human as compared with mouse N-terminal cMyBP-C (21). Regarding C0–C1 specifically, we previously measured binding levels (ratio of C0–C1 to actin) at 5 μm actin and extrapolated to the values predicted at 1 μm. In this work we find that this extrapolation is not valid (Fig. S1). Experimentally determined binding at 1 μm actin was approximately half that determined from simulation based on 1 μm. If the same holds true for C0–C1 from mouse, the then level of binding in the earlier study may be an overestimate. Finally, small technical differences in the cMyBP-C preparations may be responsible for the differences on TPA.

We hypothesize that phosphorylation of the M-domain could reduce the effects of C0–C2 on actin dynamics by one of (or a combination of) two general mechanisms: First, phosphorylation could *remove positive interactions*, eliminating/reducing interactions of the M-domain with actin that are necessary to reduce actin rotational amplitude. Secondly, phosphorylation could result in *negative interactions* with actin or other domains of cMyBP-C that have the net effect of *actively negating* the effects that their interactions have on actin dynamics. In the first hypothesis, removal of the M-domain (as in C0–C1) is predicted to have as strong an effect on the rotational amplitude as phosphorylation, or an even larger effect if positive actin interactions were provided by C2. This is not what was observed. C0–C1 resulted in a small loss in the restriction of rotational amplitude when compared with C0–C2. The restriction of C0–C2 was 22° (58° for actin alone and 36° for actin with C0–C2). For C0–C1 the restriction was 19°, only 3° different from C0–C2. PKA phosphorylation on the other hand reduced the restriction in amplitude only 12°, a difference of 10° from C0–C2. These observations support the second hypothesis where PKA phosphorylation has an active negative effect, interfering with the interaction of other domains of cMyBP-C with actin. Observations finding that phosphorylation directly alters cMyBP-C structure would also be consistent with the second hypothesis (31, 32). Both mechanisms may be operating upon PKA treatment and additional experiments will be needed to address this.

The available data indicate that changes in actin dynamics we observe in solution with isolated F-actin are also seen in skinned muscle fibers. Spectroscopic measurements probing actin dynamics in skinned fibers with fluorescent phalloidin revealed that specific conformational changes in F-actin take place during Ca^2+^-activated force development that are distinct from the transitions from the relaxed state to rigor (33). Given the cMyBP-C effects we observe in solution, we anticipate that specific effects of cMyBP-C on actin dynamics also occur in intact filaments of muscle and we are currently exploring this. The current work provides a baseline necessary for understanding the more complex details as we move forward to experiments with fully reconstituted thin filaments and intact myofilaments of skinned muscle.

### Phosphorylation mutations in the M-domain do not completely mimic phosphorylation

Numerous *in vivo* studies utilize phosphomimetic mutants of cMyBP-C to study the effects of phosphorylation of the M-domain by PKA (34–38). C0–C2 3SD (Asp for Ser substitutions at Ser-275, -284, and -304) shows very similar binding to actin in cosedimentation assays as PKA-treated or unphosphorylated C0–C2 (Figs. 2*A* and 6*A*), and this is consistent with cardiac thin filament binding of C1–C2 (22). Prior to this work, nothing was known about the relative effects on actin's structural dynamics of this N-terminal cMyBP-C binding by 3SD. Our data indicate that 3SD exerts different, intermediate effects on actin dynamics. As with phosphorylation cooperativity is abolished (*n* < 1). 3SD reduced rotational amplitude by 18%, a level lower than unphosphorylated (22%) but more than phosphorylated (12%). Thus, care should be taken extrapolating *in vivo* data utilizing the 3SD mutant as a proxy for phosphorylated cMyBP-C. A similar conclusion based on different assays and utilizing rat cMyBP-C has been reached by others (22).

### cMyBP-C in vitro phosphorylation results

**Figure 7.**
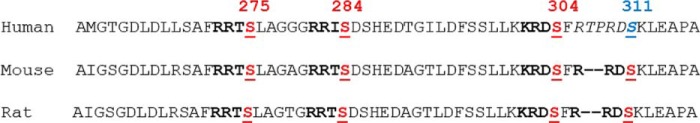
**Species-specific cMyBP-C PKA sites.** Alignment of human, mouse, and rat sequences within the M-domain that contain PKA recognition sites “R-R-*X*-S.” Four sites are found in mouse and rat but in human the final target sequence is disrupted by insertion of T-P between the R-R upstream of Ser-311.

Human cMyBP-C appears to have only three PKA recognition sites in the M-domain and this differs from mouse cMyBP-C. In control experiments, we performed LC-MS/MS on PKA-treated C0–C2 to confirm phosphorylation of the four putative target sites (Ser-275, Ser-284, Ser-304, and Ser-311). All of these serines have been demonstrated to be phosphorylated in mouse and rat cMyBP-C (39, 40). The first three serines were found to be phosphorylated, but no phosphorylation was detected on Ser-311. This specific serine in mouse and rat has been observed to be phosphorylated by others (39, 40) and by results from our lab using three distinct lines of mice (not shown). Human cMyBP-C has not been reported to be phosphorylated *in vivo* on this site and our *in vitro* results suggest that it is not a target of PKA. Examination of the sequence surrounding Ser-311 provides a likely explanation. Basic residues at positions −2 and −3 from the phosphorylated serine are strongly preferred by PKA (see Neuberger *et al.*, Ref. 41, for review). The sequence Arg-Arg-*X* or Lys-Arg-*X* (where *X* is any amino acid) precedes the four serines in mouse and rat, and three serines in human MyBP-C that are phosphorylated. Because of an apparent insertion of Thr-Pro between Arg-Arg, Ser-311 in human cMyBP-C is preceded by Pro-Arg-*X* (Fig. 7). It is likely that this change in position −3 from an arginine to proline in humans eliminates (or greatly reduces) phosphorylation by PKA as compared with rat and mouse. When these sequences are compared using an *in silico* phosphorylation program (NetPhos 3.1) the value drops from 0.82 (strong prediction of phosphorylation) to 0.44 (below the cutoff value of 0.5 for predicting phosphorylation).

The disruption in the PKA recognition sequence has been noted before (39). In this work N-terminal fragments of cMyBP-C were identified as being phosphorylated at Ser-311, although to a much-reduced level compared with Ser-275, Ser-284, and Ser-304. The levels of PKA used were quite high (25× the amount where phosphorylation of Ser-275, Ser-284, and Ser-304 reaches maximal levels in our preliminary experiments; see “Experimental procedures”). Finally, phosphorylation of Ser-311 was not observed by others (10). It remains to be determined if low-level phosphorylation of Ser-311 is physiologically relevant in humans.

We were unable to detect by LC-MS/MS or Pro-Q Diamond staining phosphorylation of recombinant human C0–C2 by GSK3β. Kuster *et al.* (23) detected phosphorylation of Ser-133 in the P/A linker by GSK3β *in vitro* using antibodies to pSer-133. In this case, antibodies did not quantitate the level of phosphorylation (*i.e.* 1 *versus* 99%). It is possible that a small fraction could be phosphorylated and not detectable by our assays. Despite not being a substrate for GSK3β phosphorylation *in vitro*, it may be one *in vivo*. Many GSK3β substrates require priming at a Ser/Thr residue four residues beyond the target. Targets not requiring priming often have a negative residue in this position (42, 43). Human cMyBP-C has a serine at position 137, but this has not been reported to be phosphorylated. *In vivo* an as of yet unidentified kinase could phosphorylate the priming residue allowing GSK3β to phosphorylate Ser-133. We note that we did not attempt Ser-133 phosphorylation in full-length cMyBP-C. To test the effects of possible phosphorylation at Ser-133, we generated the phosphomimetic Asp for Ser-133 mutation (C0–C2 S133D in Fig. 4*A*).

### Ser-133 in the P/A linker

Modification of the P/A linker Ser-133 with the phosphomimetic C0–C2 S133D had no effect on actin dynamics (Fig. 4 and Tables 1 and 2). However, it remains possible that phosphorylation of Ser-133 could have stronger effects than the S133D phosphomimetic mutation. Also, if GSK3β phosphorylation requires priming by phosphorylation at four residues after Ser-133, then there could potentially be two phosphates in this region of the P/A linker, and this could have further effects.

### cMyBP-C TPA results: Binding versus bundling

We have interpreted the TPA results to be the consequence of C0–C2 binding to F-actin. A more complicated mechanism involves C0–C2 binding to F-actin, inducing bundling of the F-actin, resulting in restricted twisting and bending components of the actin filaments. It is important to consider this alternative mechanism as F-actin has previously shown to be tightly bundled by N-terminal cMyBP-C, especially when binding is done at pH 7.5, and much less so at pH 8.0 (used in TPA) (9). We tested tight bundling with a FRET experiment under conditions used in our TPA experiments. To measure bundling we mixed F-actin labeled with IAEDANS with F-actin labeled with Fmal. If the two labeled F-actins bundle, then FRET will be observed (25). In the absence of MgCl_2_ or C0–C2, a low level of FRET was observed (1.5% FRET efficiency). Bundling the actin with 20 mm MgCl_2_ resulted in substantial FRET (35% FRET efficiency; Equation 6). Addition of C0–C2 to the levels where TPA results are saturating resulted in only a small increase in FRET (∼1.2% increase over that seen with actin alone). We conclude that the simplest explanation for our TPA results is that they can be explained by binding to actin, not subsequent bundling. Although cMyBP-C did not cause tight bundling like the MgCl_2_ positive control, it is conceivable that cMyBP-C could be causing loose bundles, especially at substoichiometric concentrations. It should also be cautioned that certain cMyBP-C interactions with actin *in vitro* could be more complicated than can be explained by Michaelis-Menten binding kinetics alone. This is evident in our comparison of binding properties at 1 *versus* 5 μm actin for C0–C2 and C0–C1. Whereas C0–C2 follows predicted Michaelis-Menten binding, the C0–C1 fragment does not (Fig. S1).

### Extrapolation to myofilament stoichiometries

**Figure 8.**
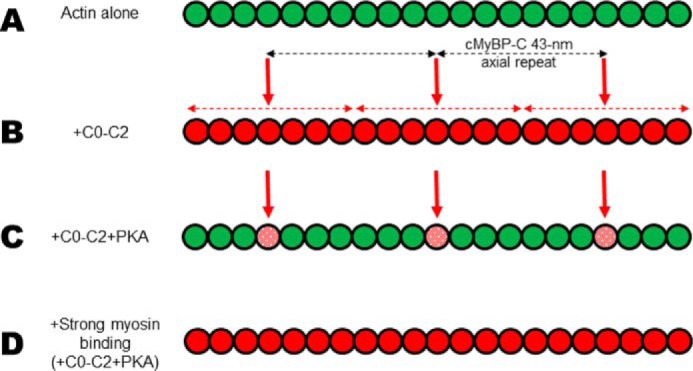
**Actin filament rotational dynamics cooperatively affected by N-terminal cMyBP-C.** Actin filaments are illustrated as a line of connected *circles* (actin monomers). Actin monomers are depicted as having large (58°) angular amplitudes (*green*) or restricted (36°) angular amplitudes (*red*). We speculate that these states are representative of weak and strong binding (to myosin) states of actin in cardiac muscle. *A*, actin alone (all *green*) exhibits weak-binding dynamics. *B*, vertical *red arrows* indicate the approximate position of N-terminal cMyBP-C binding on actin, given the 43-nm axial periodicity of cMyBP-C (horizontal *black dashed arrows*) in the C-zone of the cardiac muscle sarcomere. Given this spacing, our TPA results are consistent with the idea that adjacent unphosphorylated cMyBP-Cs could propagate structural effects of restricting dynamics all along actin residing in the C-zone. *C*, PKA treatment limits the spread of the structural effects to only its bound actin monomer. Angular amplitude is only 46° (stippled *red* in panel C) with phosphorylated C0–C2. *D*, during β-adrenergic stimulation in cardiac muscle, the increase in myosin interactions with actin would be predicted to dominate the effects on actin structural dynamics, of weak- to strong-binding states, over that of phosphorylated cMyBP-C.

Myofilament stoichiometries correlate very well with our TPA studies in solution. In the C-zone of muscle cells, cMyBP-C is estimated to be found at a ratio of 1 molecule per ∼7 actin monomers (13, 44) or ∼1:12 by other calculations (45). At this level of occupancy in our TPA experiments, effects on amplitude are much greater for unphosphorylated than phosphorylated C0–C2. Cooperativity measurements of these effects indicate that one unphosphorylated cMyBP-C impacts the structural dynamics of approximately eight monomers. The axial distribution of cMyBP-C relative to actin in the sarcomere is therefore consistent with cMyBP-C impacting actin structural dynamics of all monomers of filaments in the C-zone. This would allow for restriction of the entire C-zone actin filament. Phosphorylated cMyBP-C affects the structural dynamics of at most only the monomer it is in contact with. Graded effects on actin dynamics are possible between one and eight monomers, depending on phosphorylation. A cartoon model of the cooperative effects of N-terminal cMyBP-C on restricting actin dynamics is shown in Fig. 8.

### How actin dynamics fit into models of cMyBP-C actin binding and contractile regulation

Previous work has proposed actin-binding sites on N-terminal cMyBP-C to include domain C0 (12, 46–48), the P/A linker (only weakly) (9, 12), and the region of C1, M-domain, and C2 (9, 46, 48–50), which includes the PKA phosphorylation sites of binding regulation. Given these multiple potential contacts, our TPA results suggest that phosphorylation of the M-domain could perturb its interaction with actin and/or the interactions of other domains (C1, P/A, or C2) with actin, resulting in the loss of cooperativity and constraint of torsional dynamics. Observations finding that phosphorylation directly alters cMyBP-C structure would be consistent with this (31, 32). We and others are exploring these possibilities. In addition to contacts with actin, current models of the actin complex with N-terminal cMyBP-C suggest that interactions may involve contacts with tropomyosin (51, 52). Future spectroscopic studies of the cardiac thin filament will shed light on if and how these dynamics differ from bare actin.

The role of cMyBP-C and its phosphorylation status to modulate thin filament Ca^2+^ sensitivity has been observed both *in vitro* and *in situ*. From studies of isolated thin filaments in solution it was concluded that the N-terminal domains of cMyBP-C can bind to actin in a manner which moves the tropomyosin regulatory strand to activate and enhance contraction at low levels of Ca^2+^ and this effect is removed by phosphorylation (50, 53, 54). In studies of skinned myocardium, it was observed that cMyBP-C phosphorylation allows for faster rates of crossbridge cycling, and subsequently, because of reduced dwell time of the accelerated crossbridges, the myocardium exhibits a reduction in the Ca^2+^ sensitivity of force (55–57). The cooperative restriction of actin dynamics by N-terminal cMyBP-C that we observe could provide stabilization of the actin filament to increase the probability of myosin interacting with binding sites on it. This type of priming would be expected to be significant only during conditions of low Ca^2+^ activation, thereby increasing the Ca^2+^ sensitivity. At high Ca^2+^ levels myosin-binding sites on actin are already exposed because of tropomyosin displacement by troponin. When cMyBP-C is phosphorylated, during elevated β-adrenergic stimulation, actin dynamics are no longer restricted, because of the lack of cooperative spread of structural effects, and Ca^2+^ sensitivity is reduced. It remains to be determined how cMyBP-C and its phosphorylation may affect myosin lever arm movement and the transition from weak to strong myosin binding states, which also correlates with a transition in actin from high to low levels of rotational motion (4).

### Conclusion

During actomyosin contraction, it is essential for both myosin and actin to transition from weak- to strong-binding structural states to maintain proper cycling kinetics (1). cMyBP-C domains C0–C2 restrict the amplitude of actin's microsecond rotational dynamics, imparting these structural effects along the filament in a cooperative manner. PKA reverses this effect (partially restoring the amplitude) without dissociating the complex and exhibits no cooperativity to neighboring monomers in the filament. Phosphomimetic C0–C2 (3SD) incompletely recapitulated the effects of PKA treatment on actin TPA. The importance of the M-domain and C2 in this process is indicated by observations that C0–C1 exhibited reduced cooperativity and amplitude. Thus, phosphorylation and cMyBP-C N-terminal domains produce novel states of actin dynamics that likely impact actin-myosin crossbridge kinetics. This would allow for the tuning of the amplitudes and rates of rotational motions in response to β-adrenergic stress and other normal physiological changes.

## Experimental procedures

### Actin filament preparations

Actin was prepared from rabbit skeletal muscle by extracting acetone powder in cold water as we described in Bunch *et al.* (21). The day prior to actin-binding experiments (cosedimentation), G-actin was polymerized by the addition of MgCl_2_ to a final concentration of 3 mm. Bundled actin was removed by pelleting with a brief low-speed centrifugation (1 min, 4 °C, 3000 × *g*). Polymerized actin was dialyzed at 4 °C against actin-binding buffer (100 mm NaCl, 10 mm Tris, pH 8.0, 2 mm MgCl_2_, 0.2 mm ATP, 1 mm DTT) overnight with three changes. For phosphorescence experiments (TPA), actin was labeled at Cys-374 with ErIA (AnaSpec, Fremont, CA) (4, 19). The extent of labeling, determined by measuring dye absorbance and protein concentration, was 0.75 ± 0.05 mol dye/mol actin. The concentration of labeled actin was measured with the Pierce^TM^ BCA Protein Assay Kit (Thermo Fisher Scientific) using unmodified actin as a standard, because attached dyes had a negligible effect on this assay.

### Recombinant cMyBP-C fragment preparations

pET45b vectors encoding *Escherichia coli* optimized codons for the C0–C1 or C0–C2 portion of human cMyBP-C with N-terminal His_6_ tag and tobacco etch virus protease cleavage site were obtained from GenScript (Piscataway, NJ). In addition, C0–C1 and C0–C2 mutants were generated with S133D substitutions to test the mimicry of GSK3β-mediated phosphorylation of the P/A linker. For testing the mimicry of PKA-mediated phosphorylation of the M-domain, C0–C2 mutants were generated with Asp for Ser substitutions in three PKA sites (3SD: S275D, S284D, and S304D). Mutations were engineered in the human cMyBP-C fragments using a Q5 Site-Directed Mutagenesis Kit (New England Biolabs, Ipswich, MA). All sequences were confirmed by DNA sequencing (Eton Bioscience, San Diego, CA). Protein production in *E. coli* BL21DE3-competent cells (New England Biolabs, Ipswich, MA) and purification of C0–C1 and C0–C2 fusion proteins using His60 Ni Superflow Resin was done as described (21). cMyBP-C (cleaved of the His-tag) was then concentrated, dialyzed to 50/50 buffer (50 mm NaCl and 50 mm Tris, pH 7.5), and stored at 4 °C. Proteins were typically used for experiments within 2 weeks.

### In vitro phosphorylation of cMyBP-C

For the determination of phosphorylated serines, preliminary experiments tested phosphorylation at a wide range of PKA concentrations (0.02, 0.1, 0.5, 2.5, 13, 65, and 250 ng/μg C0–C2). Phosphorylation was monitored by in-gel staining of proteins with Pro-Q Diamond (Thermo Fisher) and staining total protein with SYPRO-Ruby (Thermo Fisher), according to the supplier's instructions. Maximal phosphorylation plateaued at 2.5 ng PKA/μg C0–C2. To ensure that all potential serines were phosphorylated, we used the sample treated with 65 ng/μg C0–C2. We excised the C0–C2 band as we described for LC-MS/MS (21) and utilized a probability-based approach for high-throughput protein phosphorylation analysis and site localization (28). The supporting information contains additional details of LC-MS/MS data processing and annotated spectra for all identified phosphopeptides (Table S1 and Fig. S2, *A–E*).

In separate preparations, C0–C2 was treated with GSK3β (G4296, Sigma) at 37 °C, for 2 h, in 10 mm imidazole, 145 mm KCl, 1 mm MgCl_2_, 2 mm EGTA, 4 mm ATP, pH 7.0. Pro-Q Diamond staining of treated C0–C2 detected no phosphorylation at any concentration up to 100×. 1× treatment is the amount required to completely phosphorylate a GSK3β substrate, based on the specific activity of 175 pmol (of a standard substrate)/min/μg GSK3β. This was repeated with separate batches of GSK3β and C0–C2. As not all phosphorylation sites are equally detected by Pro-Q Diamond, we excised the C0–C2 band for LC-MS/MS analysis.

### Actin cosedimentation assays

Actin binding by cMyBP-C fragments was determined by cosedimentation and analyzed as we have described in detail (21). Binding levels were determined at 25 °C in actin-binding buffer (100 mm NaCl, 10 mm Tris, pH 8.0, 0.2 mm ATP, and 1 mm DTT) using a constant 1 or 5 μm F-actin that was incubated with increasing amounts of C0–C2 or C0–C1 (total volume was 200 or 40 μl for binding done with 1 or 5 μm F-actin, respectively). For 1 μm binding phalloidin-stabilized ErIA-labeled F-actin was used. All cosedimentation curves were generated from at least two separate purifications of cMyBP-C (and actin). For all data points, *n* > 4 (typically *n* = 6). All reaction mixtures were made separately and not prepared in a single batch. The maximum molar binding ratio (*B*_max_) and dissociation constant (*K_d_*) values for C0–C2 binding to actin were determined by fitting the data to a quadratic model (Michaelis-Menten function) using Origin Pro 2019 computer software package through a nonlinear least-squares minimization (Levenberg Marquardt iteration algorithm). χ^2^ values of quadratic fits for all binding experiments were <0.005.

### TPA experiments and data analysis

Phalloidin-stabilized ErIA-actin was diluted in actin-binding buffer to 1 μm and increasing concentrations of unlabeled N-terminal cMyBP-C proteins were added, together with glucose oxidase to prevent photobleaching. Phosphorescence was measured at 25 °C in a TPA instrument (Fluorescence Innovations, Minneapolis, MN) as described (19). All TPA curves were generated from at least two separate purifications of cMyBP-C (and ErIA-actin). For all data points, *n* > 4. All reaction mixtures were made separately and not prepared in a single batch.

The TPA decay (anisotropy) is defined by Equation 1:

(Eq. 1) $$r\left(t\right)=\left(I_{v}\left(t\right)-GI_{h}\left(t\right)\right)/\left(I_{v}\left(t\right)+2GI_{h}\left(t\right)\right)$$

where *I_v_*(*t*) and *I_h_*(*t*) are the vertically and horizontally polarized components of the detected emission signal, and *G* is a correction factor (4, 19). TPA decays were analyzed by fitting to the sum of two exponential terms with the function in Equation 2 (4):

(Eq. 2) $$r\left(t\right)=r_{1}\exp\left(-t/\varphi_{1}\right)+r_{2}\exp\left(-t/\varphi_{2}\right)+r_{\infty }$$

where φ_1_ (fast; ∼20 μs) and φ_2_ (slow; ∼180 μs) are rotational correlation times, the corresponding amplitudes (changes in anisotropy), *r*_1_ and *r*_2_, and the final anisotropy *r*_∞_. The initial anisotropy was then calculated as *r*_0_ = *r*(0) = *r*_1_ + *r*_2_ + *r*_∞_. This method of analysis was established previously (4, 24) and was validated by comparing residuals and χ^2^ for fits with one, two, and three exponential terms, with the best fit requiring two exponential terms for actin alone and one or two exponential terms for cMyBP-C-bound actin. The overall angular amplitude of microsecond rotational motion was defined as the radius of a cone, assuming the wobble-in-a-cone model (4) (Equation 3),

(Eq. 3) $$\text{Amplitude}=\theta_{c}\left(\mu \text{s}\right)=\cos^{-1}\left[-0.5+0.5{\left(1+8{\left\{r_{\infty }/r_{0}\right\}}^{1/2}\right)}^{1/2}\right]$$

in Equation 3, the amplitude dependent on *r*_0_ and *r*_∞_ is calculated in radians which can be converted to degrees by multiplying by 180°/π. In this way, the amplitude in anisotropy units, depicted in Fig. 1*B*, is converted to an angular amplitude. Maximal flexibility corresponds to a final anisotropy of *r*_∞_ = 0, yielding a cone angle θ*_c_* = 90°, and maximal rigidity corresponds to *r*_∞_ = *r*_0_ (*i.e.* no decay) and θ*_c_* = 0 (*i.e.* no detectable rotation). The mean rate of actin filament rotational motions was defined as the inverse of the mean correlation time (Equation 4):

(Eq. 4) $$\text{Rate}=\left(r_{1}+r_{2}\right)/\left(\varphi_{1}r_{1}+\varphi_{2}r_{2}\right)$$

TPA-derived parameters (amplitude and rate) are plotted against the fractional saturation of actin binding (*v* = bound cMyBP-C molecules per actin monomer). These *v* values were determined from 1 μm phalloidin-stabilized ErIA-actin–binding assays of cMyBP-C constructs under the conditions of the TPA experiments using high-speed cosedimentation (Figs. 2*B*, 3*B*, and 4*B*).

The degree of cooperativity was determined by fitting the plot of rotational amplitude (Equation 3) or rate (Equation 4) *versus* fraction bound (*v*) to the expression of the linear lattice model (Equation 5) (4, 24–26):

(Eq. 5) $$X\left(v\right)=X_{\max}-\left(X_{\max}-X_{0}\right){\left(1-v\right)}^{n}$$

where *X*(*v*) is the observed value (angular amplitude or rate), *X*_0_ is the value for actin alone (*v* = 0), *X*_max_ is the value when actin is saturated (*v* = 1), *v* is the fraction of actin sites occupied by added protein, and *n* is the degree of cooperativity in the system, that is, the number of actin monomers affected dynamically by the binding of one cMyBP-C molecule.

As an indication of goodness of fit, χ^2^ values of the linear lattice model (cooperativity) are compared with a linear function (straight line) and are reported in Figs. 2*C*, 3*C*, and 4*C*. We note that the linear lattice model fits relatively well compared with a linear function for unphosphorylated C0–C2 (χ^2^ ∼ 4 *versus* 25) which exhibits cooperativity, whereas conditions exhibiting no significant cooperativity, such as phosphorylated C0–C2, fit similarly well to the either fitting function (χ^2^ ∼ 2 *versus* 1), as expected.

### TR-FRET testing actin bundling

Fluorescently labeled F-actin (at Cys-374) was mixed with C0–C2 (0–40 μm) or MgCl_2_ (20 mm) in actin-binding buffer and incubated for 20 min, and the fluorescence lifetimes of labeled F-actin mixtures were determined. TR-FRET of donor (IAEDANS–F-actin mixed with unlabeled F-actin) and donor-acceptor (IAEDANS–F-actin mixed with Fmal–F-actin) samples of F-actin at 5 μm (final) were measured with pulsed laser excitation and transient digitizer detection, as described (25), using a Fluorescence Lifetime Plate Reader from Fluorescence Innovations, Inc. (Minneapolis, MN), as we described earlier (21). The efficiency of energy transfer *E* was calculated from the average lifetime of the donor in presence (τ_DA_) and absence (τ_D_) of acceptor (Equation 6):

(Eq. 6) $$E=1-\left(\tau_{\text{DA}}/\tau_{\text{D}}\right)$$

### Statistics

Sample means are from four or more independent experiments. Each experiment was carried out using at least two independent protein preparations. Average data are provided as mean ± S.E. Statistical significance is evaluated by use of an unpaired *t* test. *p* values <0.05 were taken as indicating significant differences, as defined in the figure and table legends.

Data availability: The MS proteomics data have been deposited to the ProteomeXchange Consortium via the PRIDE partner repository (58) with the dataset identifier PXD015391.

## Author contributions

T. A. B. and B. A. C. conceptualization; T. A. B., R.-S. K., and V. C. L. data curation; T. A. B., R.-S. K., V. C. L., and B. A. C. formal analysis; T. A. B., R.-S. K., and V. C. L. investigation; T. A. B., R.-S. K., V. C. L., and B. A. C. methodology; T. A. B. and B. A. C. writing-original draft; T. A. B., R.-S. K., V. C. L., and B. A. C. writing-review and editing; B. A. C. supervision; B. A. C. funding acquisition.

## Supplementary Material

Supporting Information

## Footnotes

2 The abbreviations used are:

TPA: transient phosphorescence anisotropy
ErIA: erythrosine-5-iodoacetamide
P/A: Pro/Ala
TR: time-resolved
IAEDANS: 5-((((2-iodoacetyl)amino)ethyl)amino)naphtalene-1-sulfonic acid
Fmal: fluorescein-5-maleimide.
