# Supplementary material for: Human cardiac myosin–binding protein C restricts actin structural dynamics in a cooperative and phosphorylation-sensitive manner
Source: J Biol Chem. 2019 Sep 13;294(44):16228–40. doi: 10.1074/jbc.RA119.009543 (PMC6827302; doi:10.1074/jbc.RA119.009543)
Supplement: Supporting Information [file supp_294_44_16228__index.html]

Human cardiac myosin–binding protein C restricts actin structural dynamics in a cooperative and phosphorylation-sensitive manner — Human cMyBP-C N terminus actin binding and dynamics — Human cardiac myosin–binding protein C restricts actin structural dynamics in a cooperative and phosphorylation-sensitive manner — Human cMyBP-C N terminus actin binding and dynamics — Supporting Information 

# Human cardiac myosin–binding protein C restricts actin structural dynamics in a cooperative and phosphorylation-sensitive manner

## Supporting Information

- Supporting Information (to be published online) - Supplementary Materials clean version with no mark ups
- MS Word/LaTeX Source Article File - Source File Supplementary Material - Figures and Text
- Supporting Information (to be published online) - Source File Supplementary Material - Table 1
