# Supplementary material for: Human cardiac myosin–binding protein C restricts actin structural dynamics in a cooperative and phosphorylation-sensitive manner
Source: J Biol Chem. 2019 Sep 13;294(44):16228–40. doi: 10.1074/jbc.RA119.009543 (PMC6827302; doi:10.1074/jbc.RA119.009543)
Supplement: Supporting Information [file supp_RA119.009543_153139_2_supp_379132_pxmvz1.pdf]

Supplementary Figures

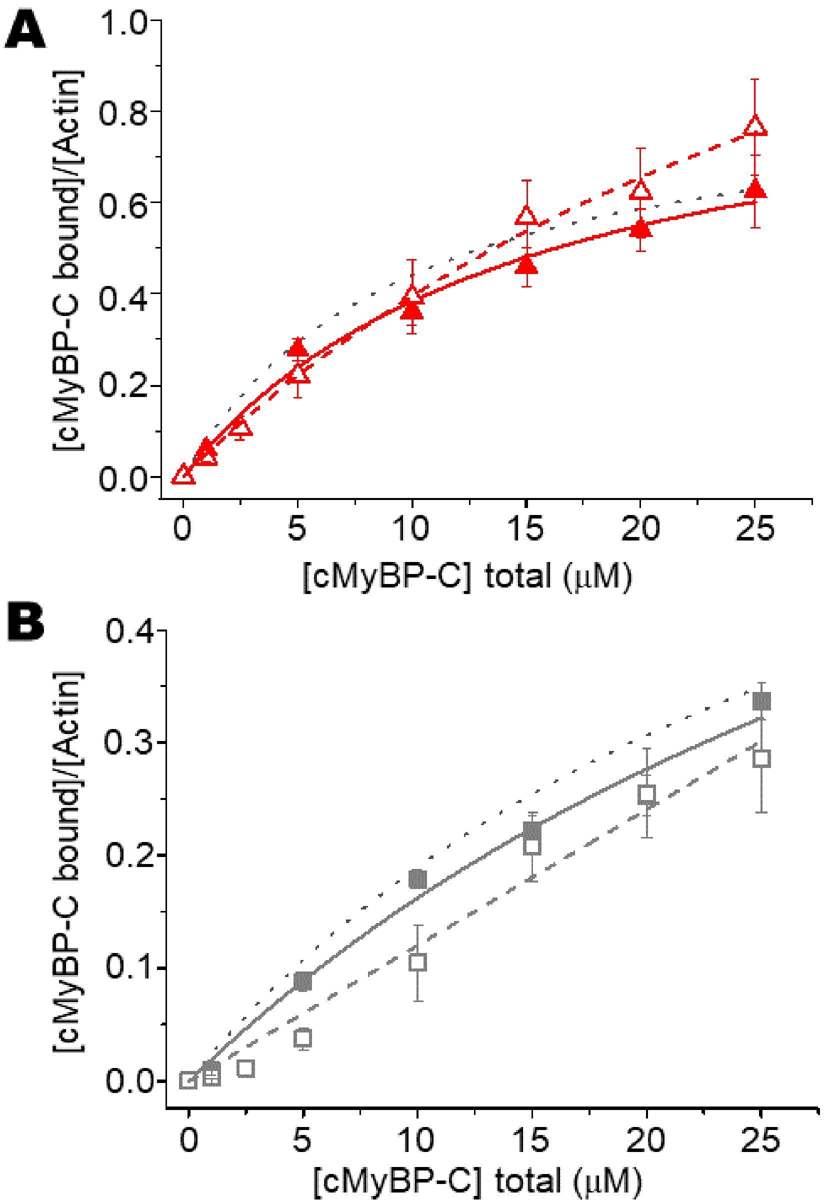

Supplementary Figure 1. Superimposed cosedimentation binding data for N-terminal cMyBP-C with unlabeled actin at 5 μM and ErIA-actin at 1 μM. Comparison of binding data at 1 μM ErIA-labeled phalloidin-stabilized F-actin (dashed lines, open shapes) and 5 μM unlabeled F-actin (solid lines, closed shapes). Also shown is the simulated binding of 1 μM actin binding extrapolated from 5 μM actin binding data. **(A)** C0-C2 (red) 1 μM binding properties were similar to the simulated values over the range of concentrations used for TPA to reach saturation of the effect (0-10 μM, main text Figure 2), whereas **(B)** C0-C1 (gray) 1 μM binding properties were significantly different over its concentration range used for TPA (0-25 μM, main text Figure 3).

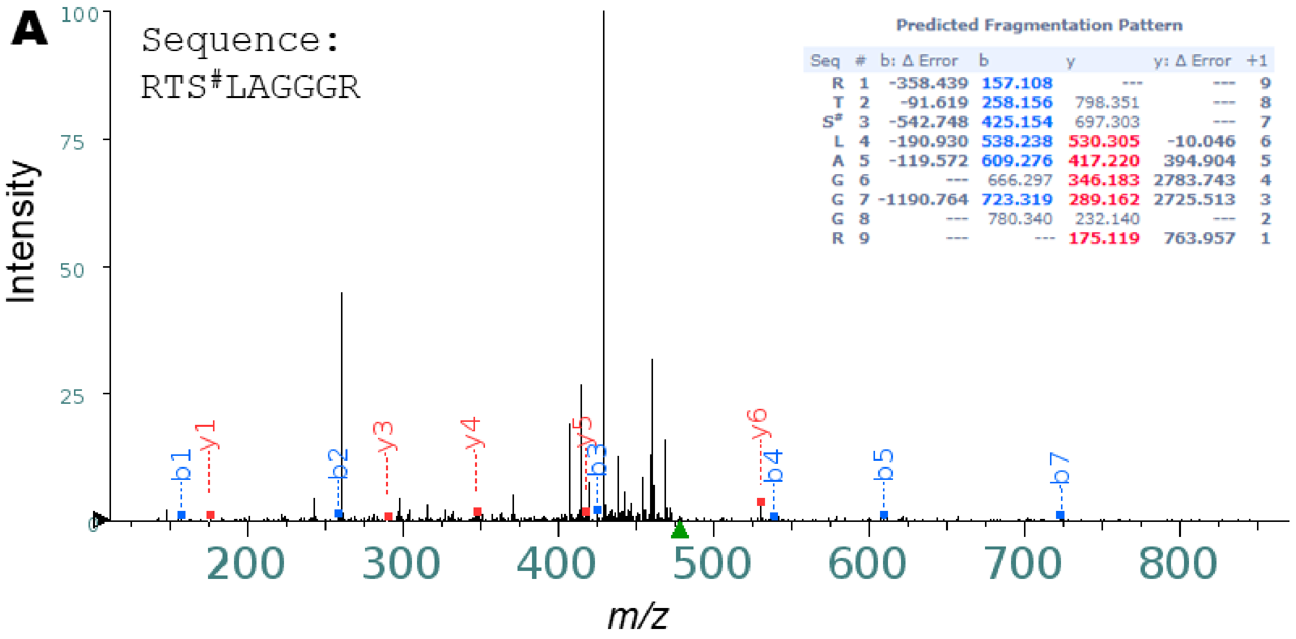

Seq	#	b: Δ Error	b	y	y: Δ Error	+1
R	1	-358.439	157.108	---	---	9
T	2	-91.619	258.156	798.351	---	8
S#	3	-542.748	425.154	697.303	---	7
L	4	-190.930	538.238	530.305	-10.046	6
A	5	-119.572	609.276	417.220	394.904	5
G	6	---	666.297	346.183	2783.743	4
G	7	-1190.764	723.319	289.162	2725.513	3
G	8	---	780.340	232.140	---	2
R	9	---	---	175.119	763.957	1

Supplementary Figure 2. Annotated mass spectra of PKA-treated C0-C2 phosphorylation sites. The green arrow indicates the theoretical *m/z* and the peak indicates the observed *m/z*. ***(A)*** **Phosphorylation site 1 (Ser275)**. The percentage of phosphorylated peptide to total peptide (phosphorylated peptide + unphosphorylated peptide) for this sequence was ~98% and demonstrates PKA phosphorylation of serine 275 in human C0-C2.

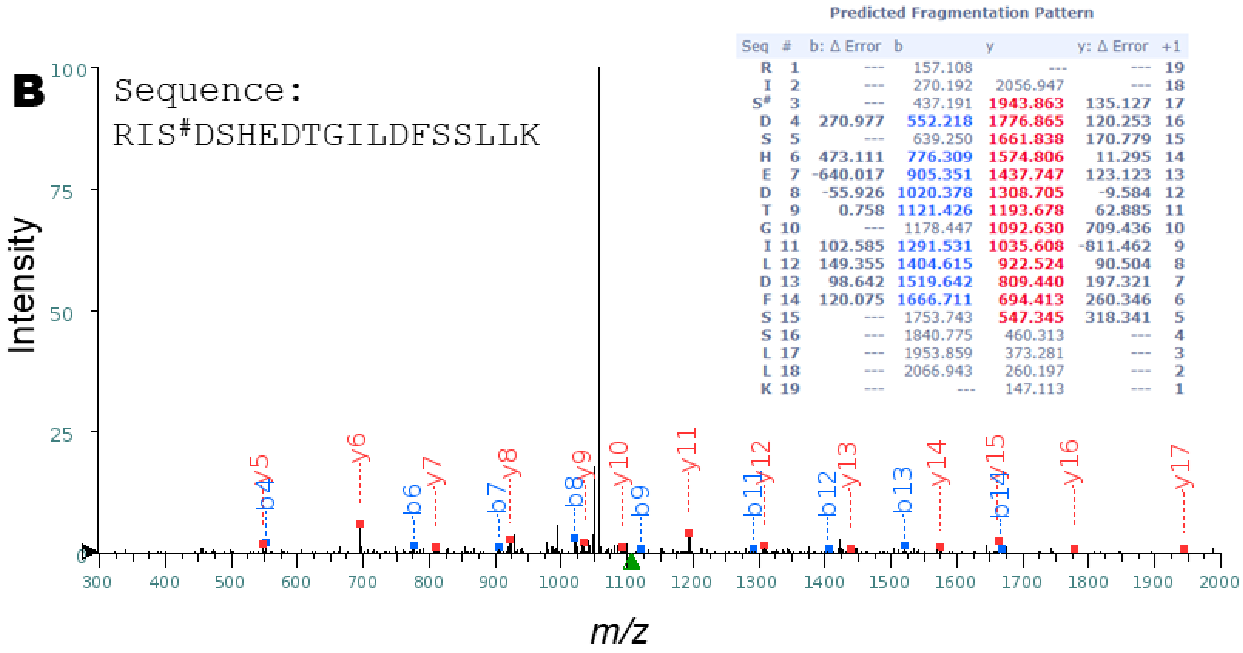

Seq	#	b: Δ Error	b	y	y: Δ Error	+1
R	1	---	157.108	---	---	19
I	2	---	270.192	2056.947	---	18
S#	3	---	437.191	1943.863	135.127	17
D	4	270.977	552.218	1776.865	120.253	16
S	5	---	639.250	1661.838	170.779	15
H	6	473.111	776.309	1574.806	11.295	14
E	7	-640.017	905.351	1437.747	123.123	13
D	8	-55.926	1020.378	1308.705	-9.584	12
T	9	0.758	1121.426	1193.678	62.885	11
G	10	---	1178.447	1092.630	709.436	10
I	11	102.585	1291.531	1035.608	-811.462	9
L	12	149.355	1404.615	922.524	90.504	8
D	13	98.642	1519.642	809.440	197.321	7
F	14	120.075	1666.711	694.413	260.346	6
S	15	---	1753.743	547.345	318.341	5
S	16	---	1840.775	460.313	---	4
L	17	---	1953.859	373.281	---	3
L	18	---	2066.943	260.197	---	2
K	19	---	---	147.113	---	1

Supplementary Figure 2. Annotated mass spectra of PKA-treated C0-C2 phosphorylation sites. The green arrow indicates the theoretical *m/z* and the peak indicates the observed *m/z*. ***(B)*** **Phosphorylation site 2 (Ser284)**. The percentage of phosphorylated peptide to total peptide (phosphorylated peptide + unphosphorylated peptide) for this sequence was ~65% and demonstrates PKA phosphorylation of serine 284 in human C0-C2.

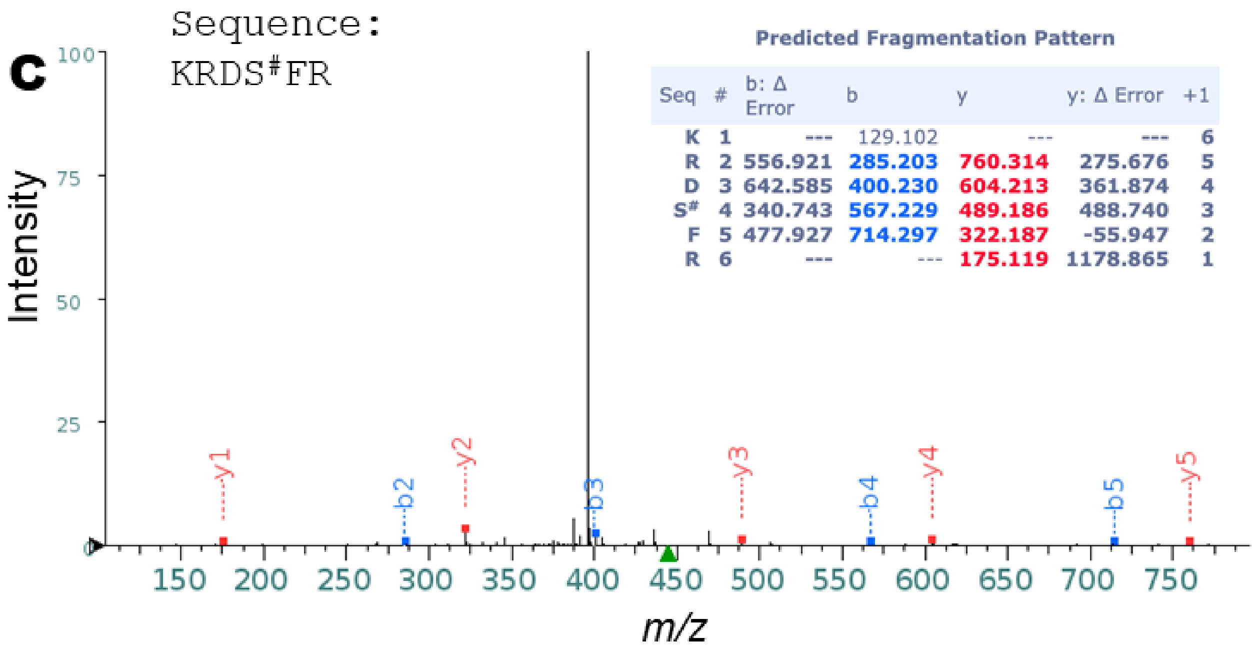

Supplementary Figure 2. Annotated mass spectra of PKA-treated C0-C2 phosphorylation sites. The green arrow indicates the theoretical *m/z* and the peak indicates the observed *m/z*. ***(C)*** **Phosphorylation site 3 (Ser304)**. The percentage of phosphorylated peptide to total peptide (phosphorylated peptide + unphosphorylated peptide) for this sequence was ~99% and demonstrates PKA phosphorylation of serine 304 in human C0-C2.

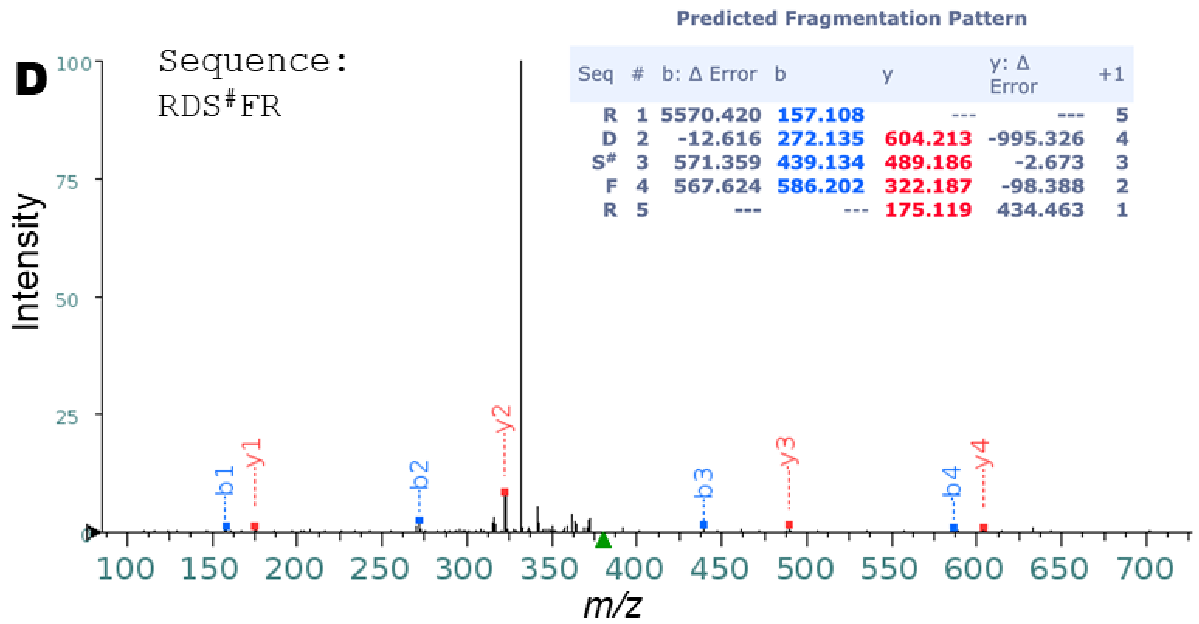

Supplementary Figure 2. Annotated mass spectra of PKA-treated C0-C2 phosphorylation sites. The green arrow indicates the theoretical *m/z* and the peak indicates the observed *m/z*. ***(D)*** **Phosphorylation site 3 (Ser304)**. The percentage of phosphorylated peptide to total peptide (phosphorylated peptide + unphosphorylated peptide) for this sequence was ~99% and demonstrates PKA phosphorylation of serine 304 in human C0-C2.

Predicted Fragmentation Pattern

+1

Seq	#	b: Δ Error	b	y	y: Δ Error	+1
D	1	---	116.034	---	---	17
S#	2	---	283.033	1964.937	---	16
K	3	---	411.128	1797.938	---	15
L	4	236.077	524.212	1669.843	---	14
E	5	59.534	653.254	1556.759	---	13
A	6	269.432	724.291	1427.717	---	12
P	7	-48.255	821.344	1356.679	---	11
A	8	134.078	892.381	1259.627	---	10
E	9	49.938	1021.424	1188.590	350.868	9
E	10	---	1150.466	1059.547	89.452	8
D	11	197.680	1265.493	930.504	23.602	7
V	12	309.900	1364.562	815.477	346.512	6
W	13	---	1550.641	716.409	108.201	5
E	14	---	1679.684	530.330	118.715	4
I	15	---	1792.768	401.287	307.521	3
L	16	---	1905.852	288.203	-300.651	2
R	17	---	---	175.119	---	1

+2

Seq	#	b: Δ Error	b	y	y: Δ Error	+1
D	1	---	58.521	---	---	17
S#	2	---	142.020	982.972	---	16
K	3	---	206.067	899.473	-947.555	15
L	4	---	262.609	835.425	1032.064	14
E	5	872.980	327.131	778.883	640.338	13
A	6	-1324.370	362.649	714.362	603.155	12
P	7	---	411.176	678.843	407.011	11
A	8	---	446.694	630.317	-1581.066	10
E	9	442.364	511.216	594.798	987.305	9
E	10	9.631	575.737	530.277	217.825	8
D	11	752.493	633.250	465.756	---	7
V	12	130.734	682.785	408.242	---	6
W	13	164.864	775.824	358.708	-119.721	5
E	14	155.481	840.345	265.668	---	4
I	15	82.294	896.887	201.147	---	3
L	16	269.419	953.430	144.605	---	2
R	17	---	---	88.063	---	1

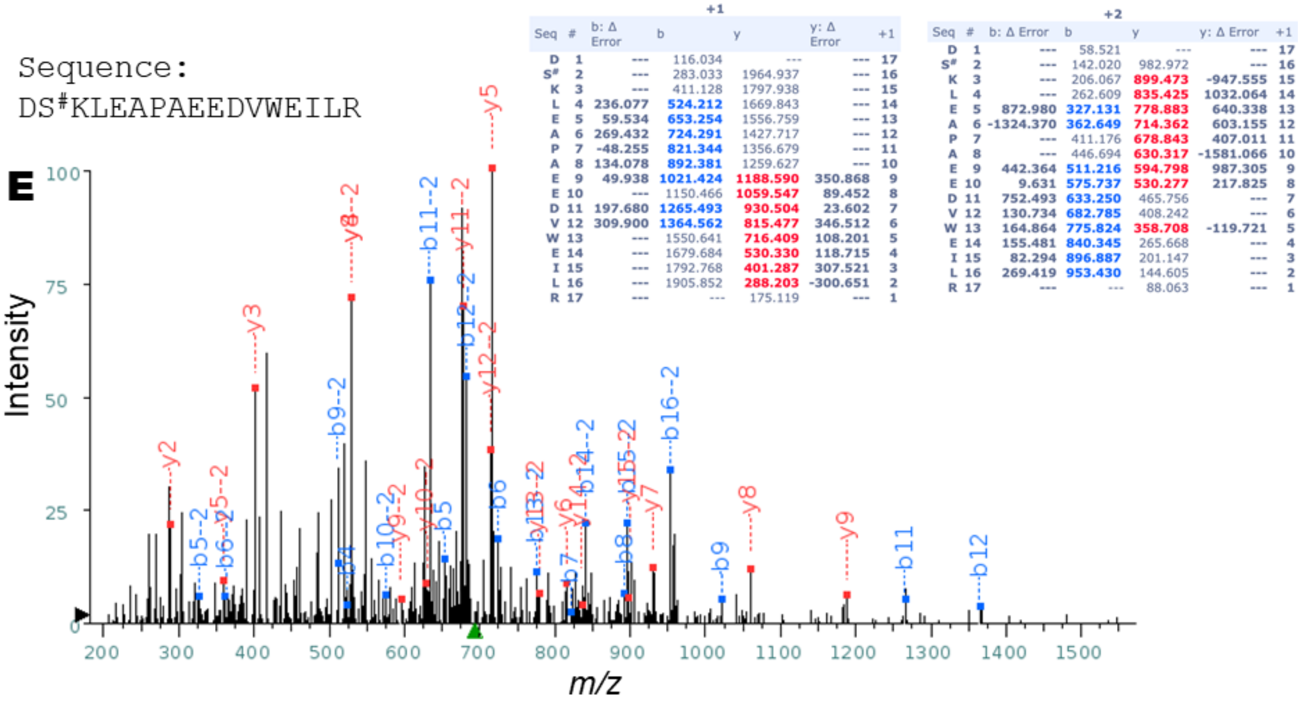

Supplementary Figure 2. Annotated mass spectra of PKA-treated C0-C2 phosphorylation sites. The green arrow indicates the theoretical *m/z* and the peak indicates the observed *m/z*. ***(E)*** **Phosphorylation site 4 (Ser311)**. The percentage of phosphorylated peptide to total peptide (phosphorylated peptide + unphosphorylated peptide) for this sequence was <0.2% and suggests PKA does not phosphorylate serine 311 in human C0-C2.

Supplementary Material

Supplementary Methods

Mass spectrometry database searching and data processing for phosphopeptide sequence assignment. LC-MS/MS proteomics data have been deposited to a publicly accessible repository, the ProteomeXchange Consortium (http://proteomecentral.proteomexchange.org) via the PRIDE partner repository (1) with the dataset identifier <PXD015391>. For mass spectrometry database searching parameters, ReAdW.exe (version 4.3.1) peaklist-generating software and SEQUEST (ver. 28, rev. 13) search engine were used. Only the target sequence was actually searched for 2 entries of the target protein and the target protein reversed. No specificity of proteases was used to generate peptides, nor was an enzyme specified for the search. However, all peptides were required to be tryptic when filtering data. The fixed modification considered was 57.0215 Da on cysteine residues and the variable modifications were methionine oxidation (15.9949 Da) and phosphorylation on serine, threonine, and tyrosine (79.9663 Da) residues. The search was done with 100 ppm mass tolerance and 1 Da for fragment ion tolerance. The threshold score/expectation value for accepting individual spectra was that the peptides must be tryptic and ppm values must be less than 10 ppm from expected, along with manual interpretation. Ascore calculation of spectra for determination of protein phosphorylation site localization was done as described (2).

REFERENCES

1. Vizcaino, J. A., Cote, R. G., Csordas, A., Dianes, J. A., Fabregat, A., Foster, J. M., Griss, J., Alpi, E., Birim, M., Contell, J., O'Kelly, G., Schoenegger, A., Ovelleiro, D., Perez-Riverol, Y., Reisinger, F., Rios, D., Wang, R., and Hermjakob, H. (2013) The PRoteomics IDEntifications (PRIDE) database and associated tools: status in 2013. *Nucleic Acids Res* **41**, D1063-1069
2. Beausoleil, S. A., Villen, J., Gerber, S. A., Rush, J., and Gygi, S. P. (2006) A probability-based approach for high-throughput protein phosphorylation analysis and site localization. *Nat Biotechnol* **24**, 1285-1292
