# Supplementary material for: Human cardiac myosin–binding protein C restricts actin structural dynamics in a cooperative and phosphorylation-sensitive manner
Source: J Biol Chem. 2019 Sep 13;294(44):16228–40. doi: 10.1074/jbc.RA119.009543 (PMC6827302; doi:10.1074/jbc.RA119.009543)
Supplement: Supporting Information [file supp_RA119.009543_153139_2_supp_379128_pxmv21.docx]

**
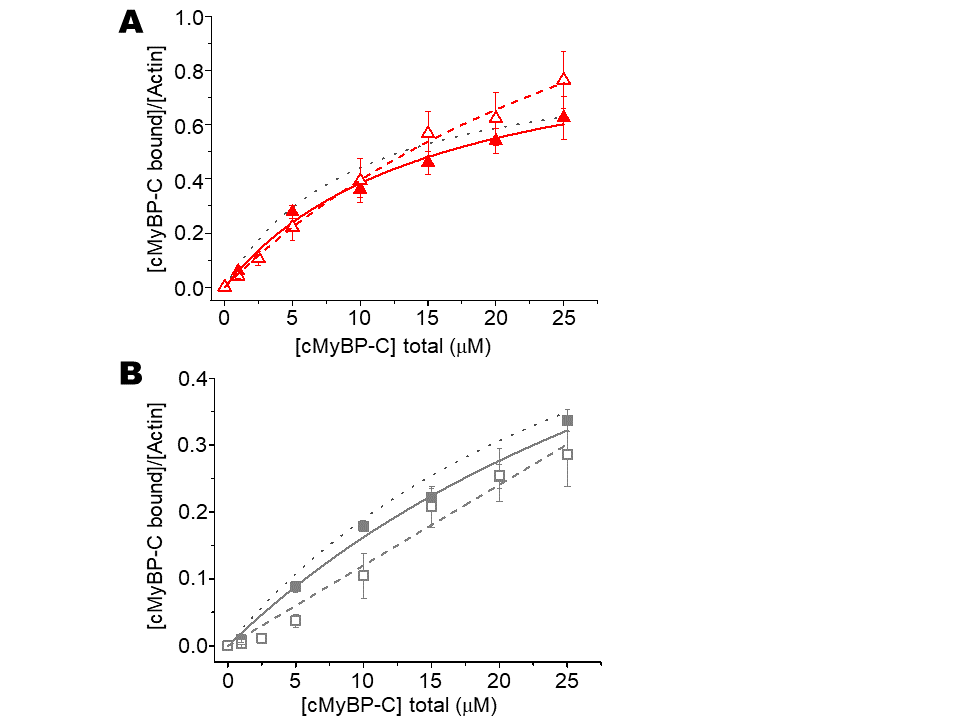
**

**Supplementary Figure 1. Superimposed** **cosedimentation binding data for N-terminal cMyBP-C with unlabeled actin at 5 μM and ErIA-actin at 1 μM.** Comparison of binding data at 1 μM ErIA-labeled phalloidin-stabilized F-actin (dashed lines, open shapes) and 5 μM unlabeled F-actin (solid lines, closed shapes). Also shown is the simulated binding of 1 μM actin binding extrapolated from 5 μM actin binding data. **(A)** C0-C2 (red) 1 μM binding properties were similar to the simulated values over the range of concentrations used for TPA to reach saturation of the effect (0-10 μM, main text Figure 2), whereas **(B)** C0-C1 (gray) 1 μM binding properties were significantly different over its concentration range used for TPA (0-25 μM, main text Figure 3).

**Supplementary Figures**

**
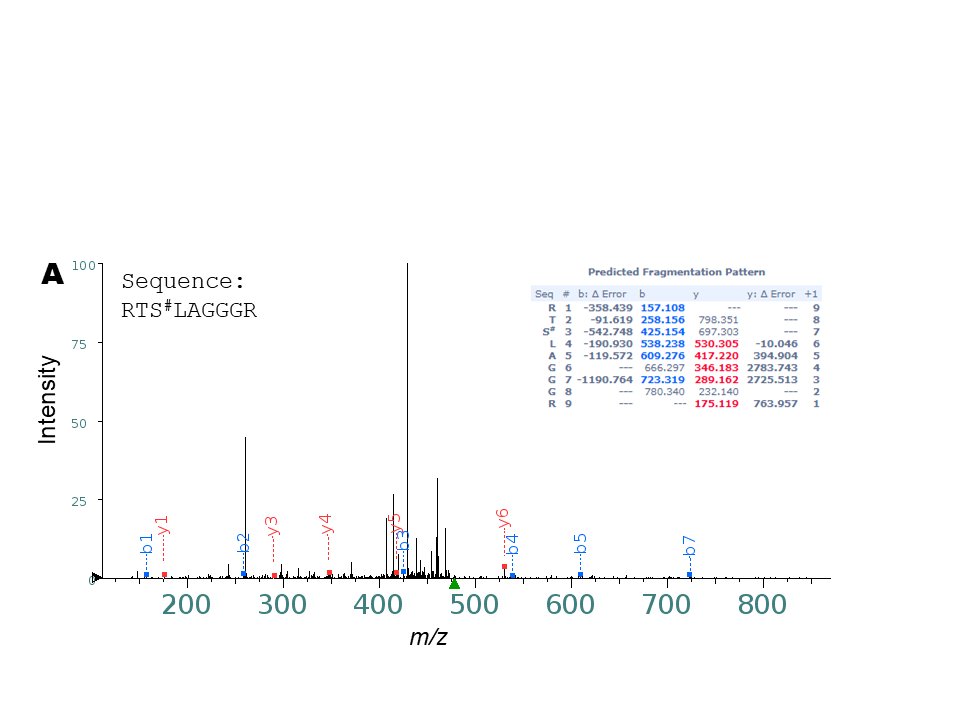
**

**Supplementary Figure 2. Annotated mass spectra of PKA-treated C0-C2 phosphorylation sites.** The green arrow indicates the theoretical *m/z* and the peak indicates the observed *m/z*. ***(A)* Phosphorylation site 1 (Ser275)**. The percentage of phosphorylated peptide to total peptide (phosphorylated peptide + unphosphorylated peptide) for this sequence was ~98% and demonstrates PKA phosphorylation of serine 275 in human C0-C2.

**
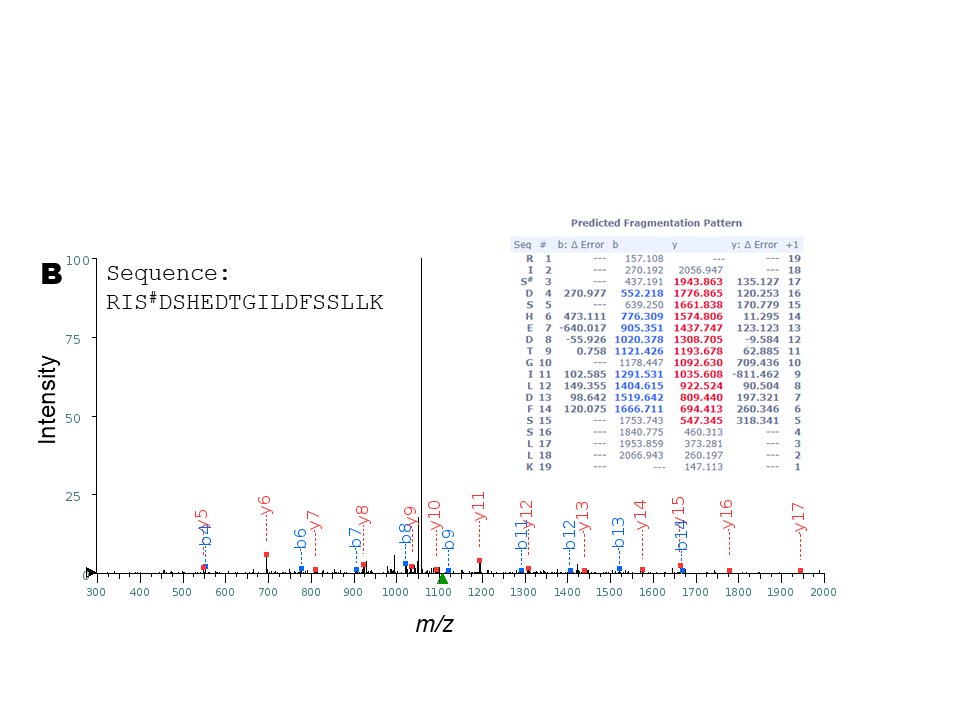
**

**Supplementary Figure 2. Annotated mass spectra of PKA-treated C0-C2 phosphorylation sites.** The green arrow indicates the theoretical *m/z* and the peak indicates the observed *m/z*. ***(B)* Phosphorylation site 2 (Ser284)**. The percentage of phosphorylated peptide to total peptide (phosphorylated peptide + unphosphorylated peptide) for this sequence was ~65% and demonstrates PKA phosphorylation of serine 284 in human C0-C2.

**
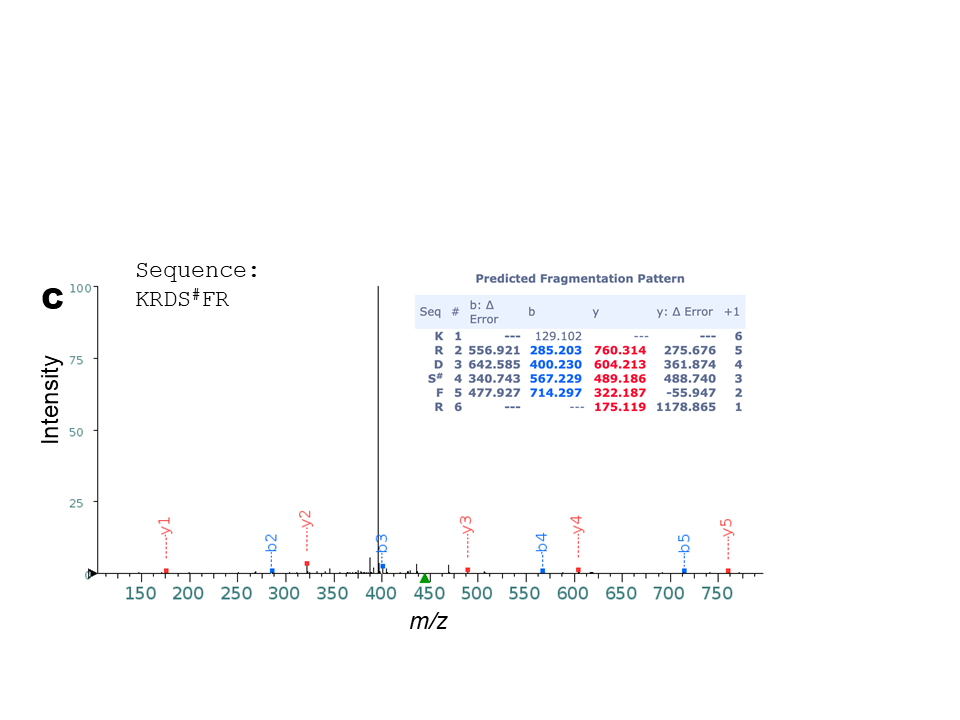
**

**Supplementary Figure 2. Annotated mass spectra of PKA-treated C0-C2 phosphorylation sites.** The green arrow indicates the theoretical *m/z* and the peak indicates the observed *m/z*. ***(C)* Phosphorylation site 3 (Ser304)**. The percentage of phosphorylated peptide to total peptide (phosphorylated peptide + unphosphorylated peptide) for this sequence was ~99% and demonstrates PKA phosphorylation of serine 304 in human C0-C2.

**
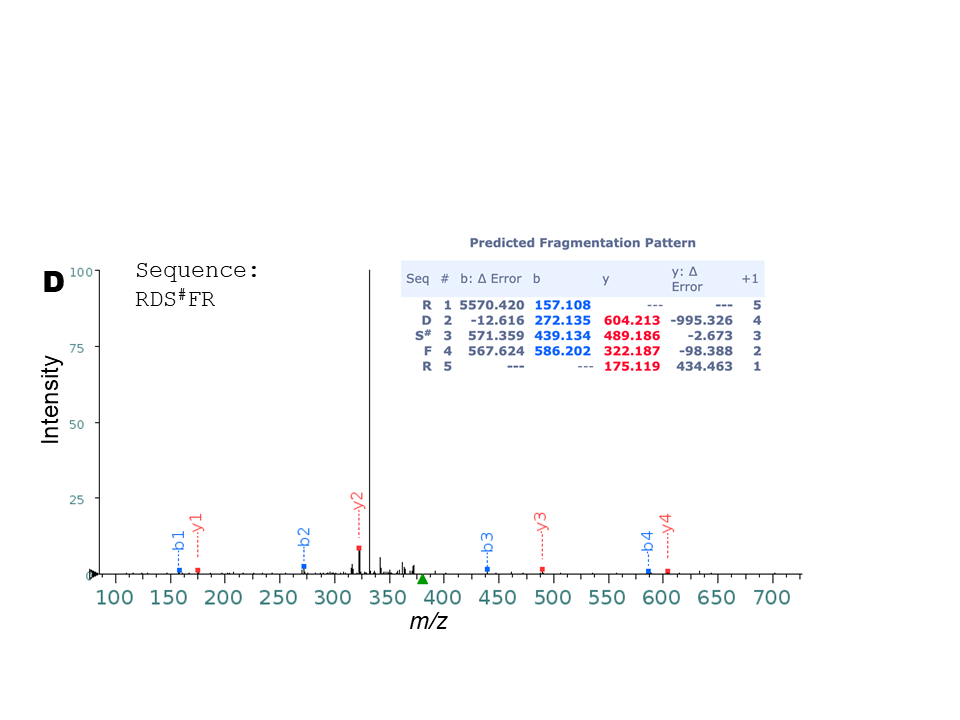
**

**Supplementary Figure 2. Annotated mass spectra of PKA-treated C0-C2 phosphorylation sites.** The green arrow indicates the theoretical *m/z* and the peak indicates the observed *m/z*. ***(D)* Phosphorylation site 3 (Ser304)**. The percentage of phosphorylated peptide to total peptide (phosphorylated peptide + unphosphorylated peptide) for this sequence was ~99% and demonstrates PKA phosphorylation of serine 304 in human C0-C2.

**
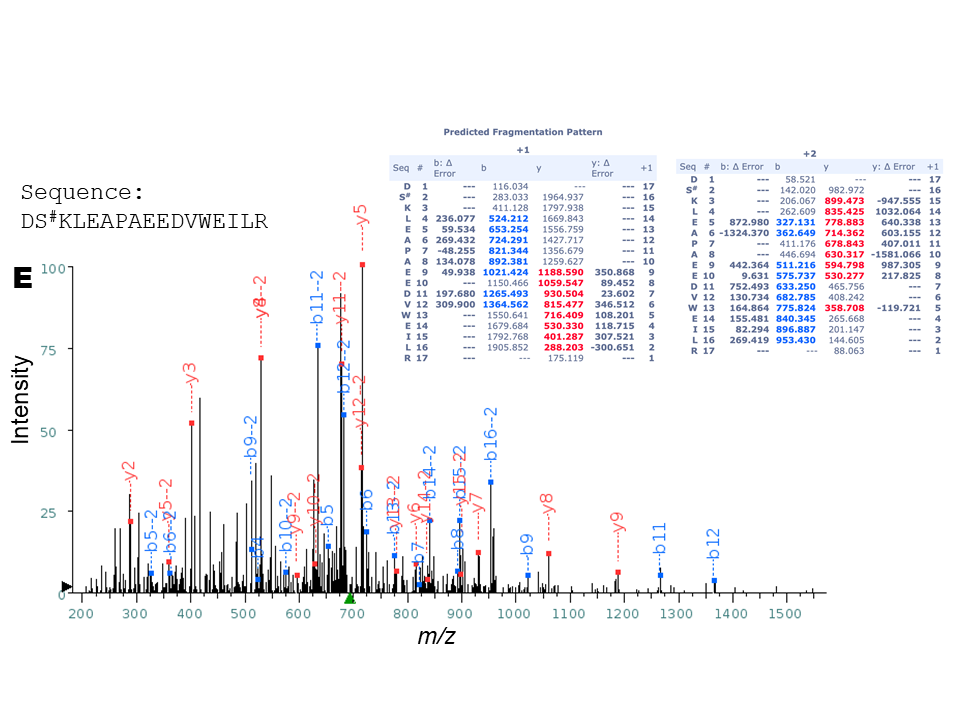
**

**Supplementary Figure 2. Annotated mass spectra of PKA-treated C0-C2 phosphorylation sites.** The green arrow indicates the theoretical *m/z* and the peak indicates the observed *m/z*. ***(E)* Phosphorylation site 4 (Ser311)**. The percentage of phosphorylated peptide to total peptide (phosphorylated peptide + unphosphorylated peptide) for this sequence was <0.2% and suggests PKA does not phosphorylate serine 311 in human C0-C2.

**Supplementary Material**

**Supplementary Methods**

***Mass spectrometry database searching and data processing for phosphopeptide sequence assignment.*** LC-MS/MS proteomics data have been deposited to a publicly accessible repository, the ProteomeXchange Consortium (<http://proteomecentral.proteomexchange.org>) via the PRIDE partner repository (1) with the dataset identifier <PXD015391>. For mass spectrometry database searching parameters, ReAdW.exe (version 4.3.1) peaklist-generating software and SEQUEST (ver. 28, rev. 13) search engine were used. Only the target sequence was actually searched for 2 entries of the target protein and the target protein reversed. No specificity of proteases was used to generate peptides, nor was an enzyme specified for the search. However, all peptides were required to be tryptic when filtering data. The fixed modification considered was 57.0215 Da on cysteine residues and the variable modifications were methionine oxidation (15.9949 Da) and phosphorylation on serine, threonine, and tyrosine (79.9663 Da) residues. The search was done with 100 ppm mass tolerance and 1 Da for fragment ion tolerance. The threshold score/expectation value for accepting individual spectra was that the peptides must be tryptic and ppm values must be less than 10 ppm from expected, along with manual interpretation. Ascore calculation of spectra for determination of protein phosphorylation site localization was done as described (2).

**REFERENCES**

1. Vizcaino, J. A., Cote, R. G., Csordas, A., Dianes, J. A., Fabregat, A., Foster, J. M., Griss, J., Alpi, E., Birim, M., Contell, J., O'Kelly, G., Schoenegger, A., Ovelleiro, D., Perez-Riverol, Y., Reisinger, F., Rios, D., Wang, R., and Hermjakob, H. (2013) The PRoteomics IDEntifications (PRIDE) database and associated tools: status in 2013. *Nucleic Acids Res* **41**, D1063-1069

2. Beausoleil, S. A., Villen, J., Gerber, S. A., Rush, J., and Gygi, S. P. (2006) A probability-based approach for high-throughput protein phosphorylation analysis and site localization. *Nat Biotechnol* **24**, 1285-1292
