# Supplementary material for: Human cardiac myosin–binding protein C restricts actin structural dynamics in a cooperative and phosphorylation-sensitive manner
Source: J Biol Chem. 2019 Sep 13;294(44):16228–40. doi: 10.1074/jbc.RA119.009543 (PMC6827302; doi:10.1074/jbc.RA119.009543)
Supplement: Supporting Information [file supp_RA119.009543_153139_2_supp_393019_pxmv81.docx]

**Supplementary Table 1:**  **Protein phosphorylation analysis and site localization of PKA-treated human C0-C2 from LC-MS/MS**

| Peptide | Site | % Phosphorylated | Precursor charge (z) | Observed *m/z* | Theoretical *m/z* | Site Score (Ascore) |
| --- | --- | --- | --- | --- | --- | --- |
| R.RTS#LAGGGR.R | 276 | 97.8 | 2 | 477.72663 | 477.72947 | 24.4 |
| R.RIS#DSHEDTGILDFSSLLK.K | 285 | 64.7 | 3 | 738.35149 | 738.3544 | 30.8 |
| K.KRDS#FR.T | 305 | 98.5 | 2 | 444.70657 | 444.70801 | 1000 |
| K.RDS#FR.T | 305 | 98.5 | 2 | 380.65959 | 380.66053 | 1000 |
| R.DS#KLEAPAEEDVWEILR.Q | 312 | <0.2*** | 3 | 693.99058 | 693.9927 | 1000 |

LC-MS/MS data from all identified phophopeptide sequences in sample. ***Very low abundance ratio of phosphorylated peptide to total peptide of identified site 312 sequence [% Phosphorylated = S#/(S#+S), where S# is the abundance of the phosphorylated peptide and S is the abundance of the unphosphorylated peptide of the PKA-treated sample] in C0-C2 samples treated with saturating PKA. Site score values ≥19 are considered confidently assigned with near certainty (> 99%) and values listed as 1000 denotes that there are no other possible sites to assign the phosphate and the assignment is 100% unequivocal (2).
